# Habitat loss exacerbates pathogen spread: An Agent-based model of avian influenza infection in migratory waterfowl

**DOI:** 10.1371/journal.pcbi.1009577

**Published:** 2022-08-18

**Authors:** Shenglai Yin, Yanjie Xu, Mingshuai Xu, Mart C. M. de Jong, Mees R. S. Huisman, Andrea Contina, Herbert H. T. Prins, Zheng Y. X. Huang, Willem F. de Boer

**Affiliations:** 1 College of Life Science, Nanjing Normal University, Nanjing, China; 2 Wildlife Ecology and Conservation Group, Wageningen University, Wageningen, The Netherlands; 3 The Finnish Museum of Natural History, University of Helsinki, Helsinki, Finland; 4 Quantitative Veterinary Epidemiology Group, Wageningen University, Wageningen, The Netherlands; 5 Department of Integrative Biology, University of Texas at Austin, Austin, Texas, United States of America; 6 Department of Animal Sciences, Wageningen University, Wageningen, The Netherlands; Johns Hopkins University, UNITED STATES

## Abstract

Habitat availability determines the distribution of migratory waterfowl along their flyway, which further influences the transmission and spatial spread of avian influenza viruses (AIVs). The extensive habitat loss in the East Asian-Australasian Flyway (EAAF) may have potentially altered the virus spread and transmission, but those consequences are rarely studied. We constructed 6 fall migration networks that differed in their level of habitat loss, wherein an increase in habitat loss resulted in smaller networks with fewer sites and links. We integrated an agent-based model and a susceptible-infected-recovered model to simulate waterfowl migration and AIV transmission. We found that extensive habitat loss in the EAAF can 1) relocate the outbreaks northwards, responding to the distribution changes of wintering waterfowl geese, 2) increase the outbreak risk in remaining sites due to larger goose congregations, and 3) facilitate AIV transmission in the migratory population. In addition, our modeling output was in line with the predictions from the concept of “migratory escape”, i.e., the migration allows the geese to “escape” from the location where infection risk is high, affecting the pattern of infection prevalence in the waterfowl population. Our modeling shed light on the potential consequences of habitat loss in spreading and transmitting AIV at the flyway scale and suggested the driving mechanisms behind these effects, indicating the importance of conservation in changing spatial and temporal patterns of AIV outbreaks.

## Author summary

What are the possible consequences of extensive habitat loss on the spread and transmission of avian influenza viruses (AIVs)? We used a logistic regression model to select the suitable habitats of Greater white-fronted goose *Anser albifrons* in the East Asian-Australasian Flyway, which has extensive habitat loss and seasonal passing waterfowl population. We treated these habitats as sites to construct 6 fall migration networks by removing sites in order of habitat loss (i.e., site removal). We then simulated geese migration in these networks and explored the impacts of habitat loss on AIV spread and transmission. We found that extensive habitat loss can cause relocation of the geese and outbreaks and promote the AIV spread and transmission. Our modeling outputs also suggested a “migratory escape” pattern for changing the spatial and temporal pattern of infection prevalence in the population. Overall, our study showed that habitat loss can affect AIV spread and transmission, suggesting the importance of habitat conservation in changing the spatial and temporal pattern of disease prevalence.

## Introduction

Migration is a common animal behavior in nature, accompanied by large ecological effects [1,2]. As migrants, especially birds, are hosts of pathogens, their seasonal migration contributes to pathogen spread and associates with the spatio-temporal patterns of the infection dynamics [3,4]. Well-known examples include the interactions between birds migration and the emergence of the West Nile virus [5], nematodes [6], malaria [7], Lyme *borreliosis* [8], and avian influenza viruses (AIVs) [9]. For example, the West Nile virus was spread throughout the North America within 4 years after its first detection in the US, mainly driven by seasonal bird migration [5]. Better understanding the effect of host migration on pathogen spread and transmission is of great importance for the prediction and prevention of disease emergence and spread.

Changes in habitat availability can fundamentally alter bird migration, which subsequently affects the interactions between pathogens and hosts [3]. For example, changes in habitat availability (and quality) may alter migration distance, duration or even establish sedentary populations [10–12]. Bird migration is flexible in responding to changes in habitat availability by changing migration timing, routes, or by occupying new habitats [13,14]. These responses can affect disease spread and transmission by changing birds’ distribution and congregation (i.e., local numbers and densities), but the specific relationships between pathogen distribution and changes in migration patterns of hosts remain unclear.

Avian influenza viruses (AIVs) are well-known for their rapid global spreading and frequent outbreaks over the past decades [15–17]. The transmission in the wild waterfowl populations is mainly attributed to the seasonal migration and congregation of the waterfowl [16]. First, some migratory waterfowl can migrate over long-distance while infected with AIVs [18,19]. Second, the seasonally formed waterfowl congregations can facilitate AIVs transmission by increasing direct and indirect contacts between birds [20–22].

The East Asian-Australasian Flyway (EAAF) has been identified as a high-risk area for AIVs outbreak [20,23,24] due to the considerable amounts of passing waterfowl and dense congregation along the flyway [25,26]. For instance, on the migratory route of Swan goose *Anser cygnoides*, there were more than 20 outbreaks of highly pathogenic AIVs between 2004 and 2017 [24]. Especially, the Yangtze River floodplain, the wintering region for many waterfowl species, has become a well-known region for AIVs outbreaks [9,27].

Anthropogenic disturbances such as urban development and land reclamation have caused rapid habitat loss and degradation in the EAAF, especially in the Yangtze River floodplain [28,29]. Consequently, migratory waterfowl changed their distribution by using alternative habitats for refueling and wintering [30,31]. For example, previous field surveys suggested that at least 27 waterbird species have changed the distribution [32] and concentrated in fewer remaining habitats [33,34].

Greater white-fronted goose *Anser albifrons* is one of the main waterfowl hosts of AIVs, and an important vector for the AIVs spread and transmission in the EAAF [17]. Since their migration has been well documented by previous GPS telemetry tracking studies [35,36], The Greater white-fronted goose is an ideal model species to examine the potential consequences of habitat loss on AIVs spread and transmission in the EAAF. They breed as north as the Lena Delta in Siberia and seasonally migrate to their wintering grounds in the Yangtze River floodplain, south Japan and South Korea [13,37]. Their suitable habitats form a relatively narrow-long migration corridor, making the geese distribution sensitive to habitat loss [13]. Extensive habitat loss in the wintering region may cause the Greater white-fronted goose to relocate and concentrate, i.e., increase in numbers in remaining habitats [34], thereby affect AIVs spread and transmission. Thus, we expect that the outbreak risks change spatially and temporally under the influence of habitat loss [38], with the remaining habitats having a greater risk of AIV outbreak.

In this study, we applied 6 different scenarios of habitat loss to fall migration networks of the Greater white-fronted goose in the EAAF, integrated with an agent-based model (ABM) to simulate the migration of waterfowl and with a susceptible-infected-recovered model (SIR) to simulate the virus spread among habitats and the transmission in the population. We explored the possible consequences of habitat loss on the spread and transmission of AIV. More specifically, we aimed to answer three questions: (1) How does habitat loss change the spatial distribution of AIV outbreaks under influence of changes in the distribution of the Greater white-fronted goose? (2) Does habitat loss facilitate AIV spread in remaining habitats? (3) Can habitat loss increase virus transmission in a migrating population?

## Results and discussion

### Geese and outbreaks relocation

**Fig 1:**
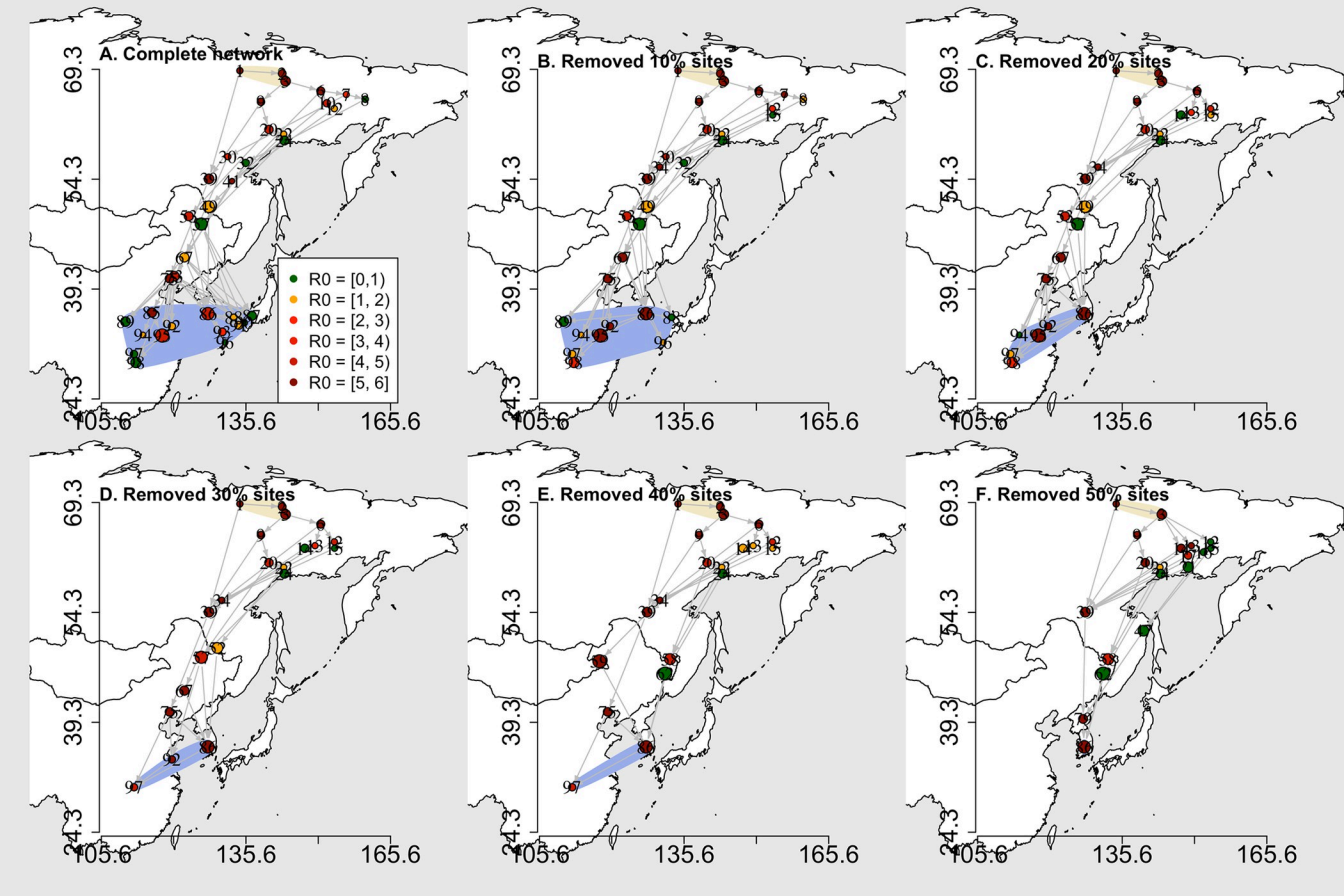
The visited migration networks generated by the simulated geese movement according to the agent-based model. A) the complete network, and B-F) the network scenarios of 10%, 20%, 30%, 40% and 50% site removal. Dots are sites visited by geese, colors represent the maximum R_0_ that occurred at sites during the simulation, and numbers are site IDs. Yellow and blue shadows depict breeding and wintering regions. The base layer maps were generated in R environment using the “maps” package (Original S code by Richard A. Becker, Allan R. Wilks. R version by Ray Brownrigg. Enhancements by Thomas P Minka and Alex Deckmyn. (2016). maps: Draw Geographical Maps. R package version 3.1.1. http://CRAN.R-project.org/package=maps).

We removed sites in order of the descending area of habitat loss to generate network scenarios (see Method). Generally, site removal causes the relocations of wintering geese and AIV outbreaks (i.e., sites with R_0_>1). As the habitat loss was mainly concentrated at the Yangtze River floodplain (Fig B in S1 Appendix), the wintering region drastically shrunk in the scenarios of more than 20% of sites removal (Fig 1C–1F). Thus, the migratory geese and AIV outbreaks were confined to smaller geographic areas. Particularly in the extreme scenario where 50% of sites were removed (Fig 1F), the wintering geese and outbreaks were restricted in the area above 35.9° N where the last wintering site was located (site ID = 86; also see Fig C in S1 Appendix for geese distribution at each site in each time step). In reality, the severity of habitat degradation and loss in the Yangtze River floodplain has been called for attention since the 1990s, these processes are still ongoing [29,30]. Although we are here theoretically simulating such an extreme level of site removal to explore the potential consequences, the northwards relocation of wintering geese can happen before extensive sites disappear, especially for species that are sensitive to changes in habitat availability. For example, a field survey suggested Bean goose *Anser fabalis*, Ruddy shelduck *Tadorna ferruginea*, and Red-breasted merganser *Mergus serrator* had started to shift their wintering habitats northwards already a decade ago in the Yangtze River floodplain [32].

The relocation of wintering Greater white-fronted geese caused the northwards shift of AIV outbreaks. Other waterfowl species that have similar narrow-long migration corridors such as Bean goose, Lesser white-fronted goose *Anser erythropus*, Tundra swan *Cygnus columbianus*, Common teal *Anas crecca*, and Northern pintail *Anas acuta* may lead to similar outbreak relocation in the EAAF [13]. However, waterfowl species such as Greylag goose *Anser anser* and Swan goose may have different effects on the AIV spread because their migration corridors are shaped differently (i.e., wide-short shaped corridor) [13]. Thus, future studies could consider multiple migratory species to explore the effects of their interactions on AIV spread.

### AIV spread in sites

**Fig 2:**
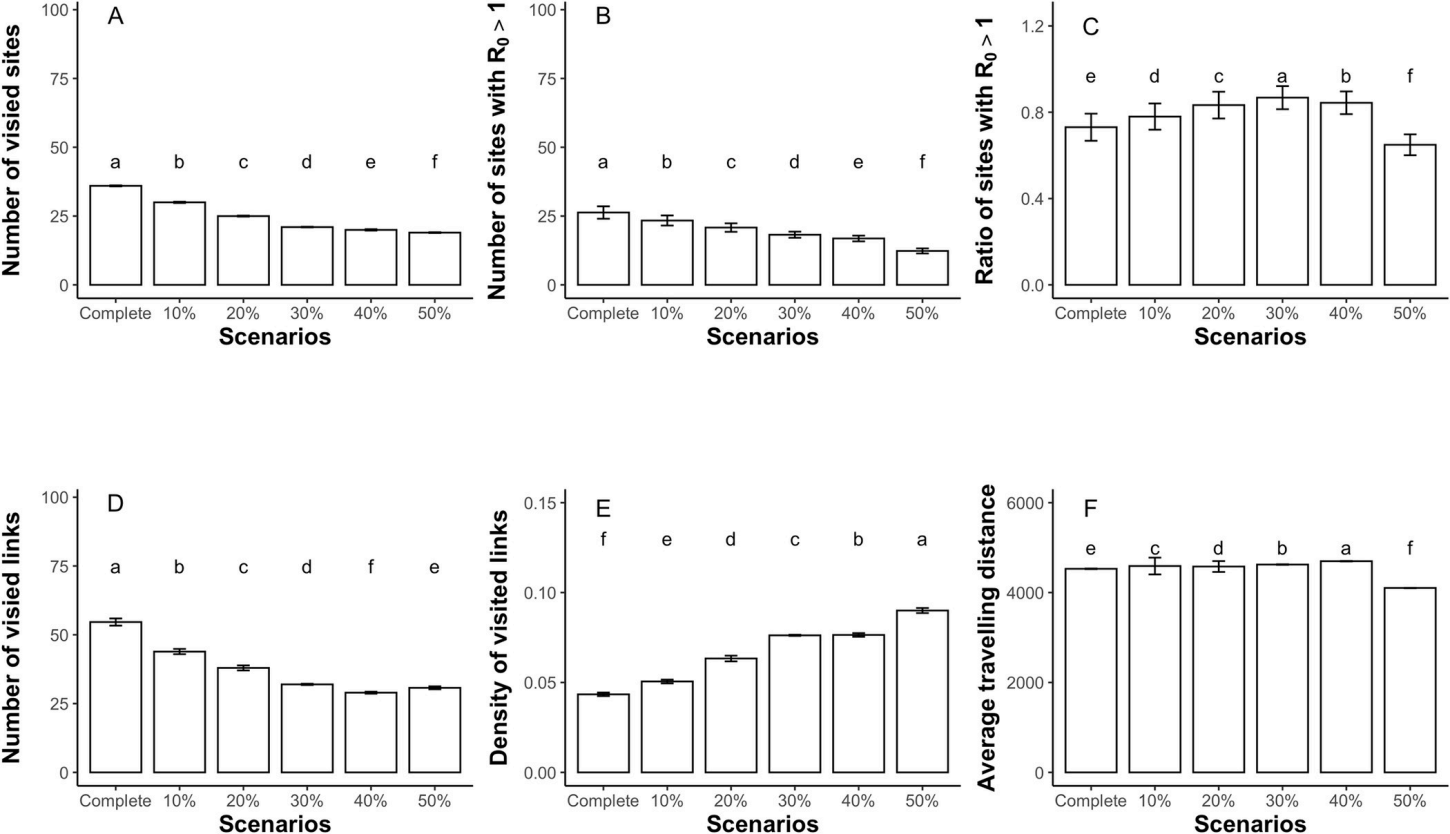
The basic metrics (mean ± SD) of the visited networks under increasing site removal. On the x axis, from left to right, the scenario of the complete network, and the network scenarios of 10%, 20%, 30%, 40% and 50% site removal. The lowercase annotations indicate the statistic difference according to Kruskal-Wallis tests (p < 0.05).

**Fig 3:**
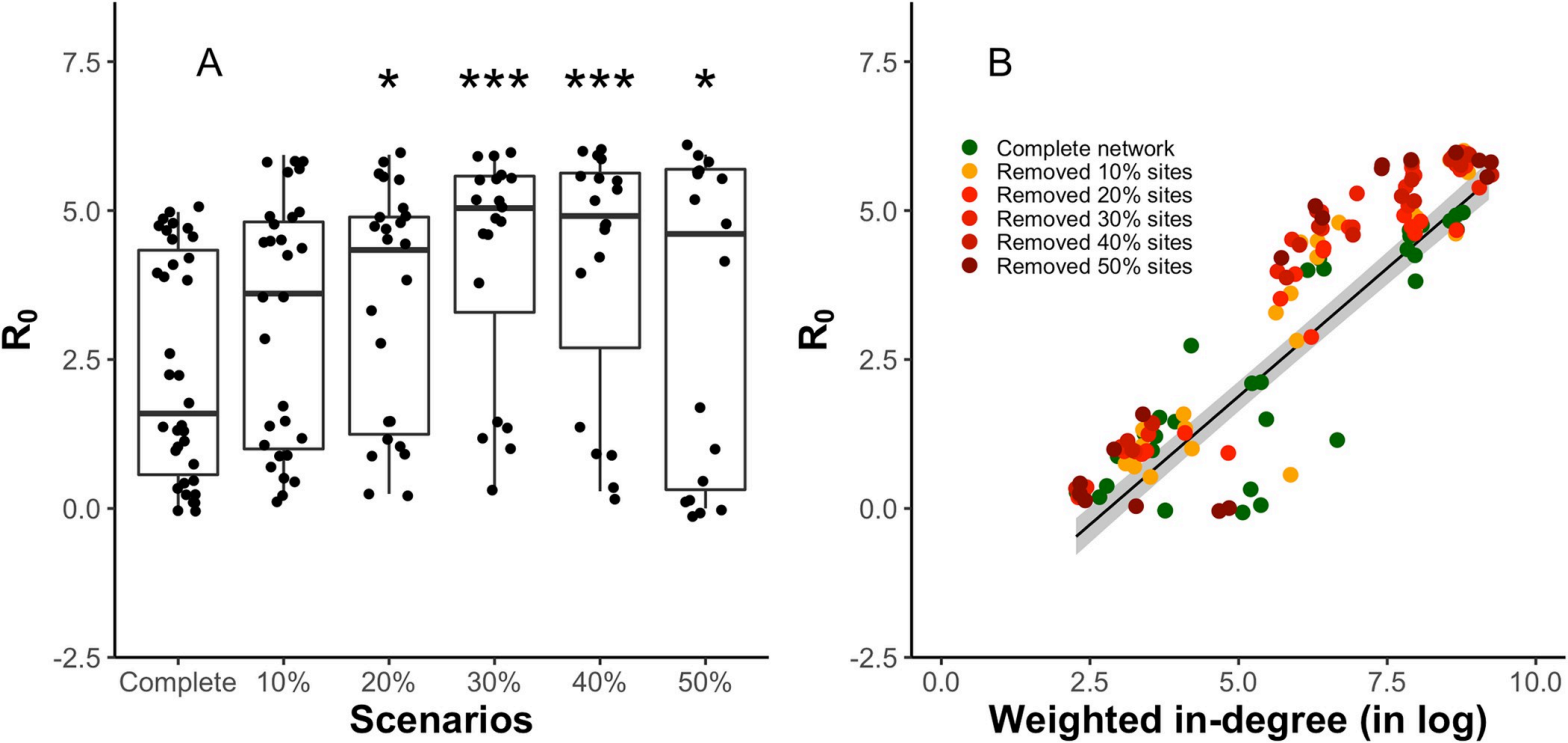
**The effects of A) increasing sites removal and B) weighted in-degree on the basic reproduction number R**_**0**_ **at each site.** A) x-axis labels are the scenario of the complete network, and network scenarios of 10%, 20%, 30%, 40% and 50% removal of sites; B) black line represents the GLM fit, and grey shaded area represent the 95% confidence interval. The asterisk represents the levels of statistical difference (* for p<0.05, and *** for p<0.001), compared to the complete network scenario. Colored dots are the maximum R_0_ values generated by agent-based model simulations under different network scenarios.

Site removal caused migratory geese to visit fewer sites in the migration networks, accompanied by fewer sites with AIV outbreak (Fig 2A and 2B), however, it also caused a higher outbreak risk in remaining sites (Figs 2C and 3A). Although the geese can use alternative sites for migration (e.g., sites ID = 13, 14, 34, 58, 59, etc., Fig 1, and also see Table A in S1 Appendix for changes in visiting geese and R_0_ at each site), they also formed larger congregations in the remaining sites (e.g., sites ID = 12, 39, 67, 86 and 97, etc., Table A and Fig C in S1 Appendix). Thus, the geese relocation and larger congregations in remaining sites promoted AIV spread and transmission, increasing outbreak risk, and even converted low-risk sites to high-risk sites (e.g., sites ID = 13, 14, 34, 57, etc., Fig 1 and Table A in S1 Appendix). In fact, waterfowl can occupy alternative habitats when their prime habitat is no longer available [10], increasing congregations at these alternative habitats between scenarios [34]. For example, about 95% of the Swan goose population is nowadays confined to only three of their major habitats [33], other species such as the Greylag goose, Tundra bean goose *Anser serrirostris*, Lesser white-fronted goose, and Greater white-fronted goose are showing similar trends, with larger congregations in fewer remaining prime habitats [34].

The basic reproduction number R_0_ increased with weighted in-degree (Fig 3B; also see Fig D in S1 Appendix for the regressions for wintering sites and non-wintering sites separately), indicating that highly connected sites with more arriving geese have a larger AIV outbreak risk, because they are more likely to receive infected geese. In reality, the well-connected habitats with better connections and more visiting waterbirds are commonly recognized as crucial habitats for foraging and resting of migratory waterbirds [39,40]. Well-known examples include Delaware Bay and Poyang Lake [20,41], which are also hotspots for AIV outbreaks [20]. Field surveillance at crucial habitats is important for understanding the epidemiological dynamic in wild waterbirds [15]. However, many habitats, especially those located in remote areas, received little attention [15,42], largely because their importance is not sufficiently evaluated. Recent studies used network analysis, integrated with GPS telemetry, to study the importance of remote habitats in terms of their connectivity to other sites and waterbirds use [40,43]. Future studies can implement these approaches to evaluate the importance of these remote habitats and identify potential AIVs hotspots.

Furthermore, our simulations also suggested that, although the geese migrated within smaller networks due to increasing level of site removal (Fig 2A and 2D and Fig B in S1 Appendix), the resulting migration generated networks with higher link densities (Fig 2E), indicating that connections among the remaining sites is enhanced, which can contribute to more rapid AIV spread and transmission [3,44–46]. Our simulation outputs suggested that local habitat loss can trigger changes in geese distribution and AIV spread at flyway scale, so planning habitat conservation at flyway scale is required when formulating efficient control actions.

### Virus transmission in population

**Fig 4:**
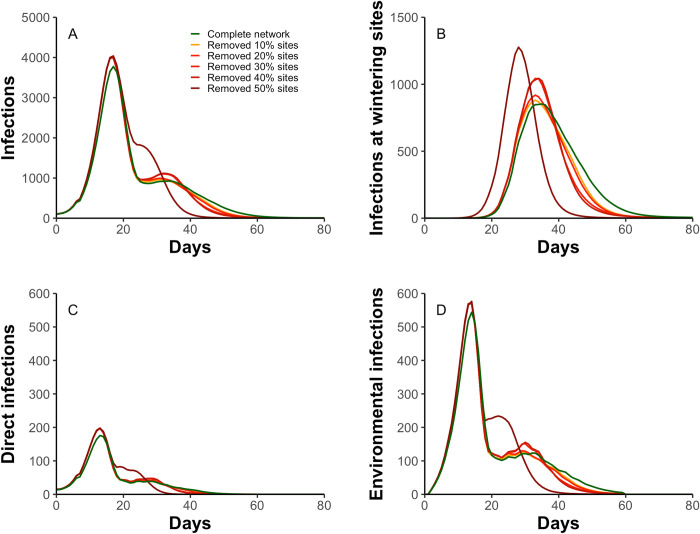
The dynamics of infection prevalence in the migratory population. A) number of infections; B) number of infections at the wintering sites; C) number of infections caused by direct transmission; D) number of infections caused by indirect environmental transmission. Line colors represent the infection dynamics under different network scenarios.

Site removal changed the temporal infection patterns and the prevalence estimates in the migratory population (Fig 4). Specifically, site removal caused the AIV to transmit faster between geese due to larger congregations, increasing virus accumulation in the environment (Fig E in S1 Appendix for the virus accumulation) so that the indirect transmission increased, which contributed more to AIV prevalence (Fig 4C and 4D). Particularly in the extreme scenario, the removal of wintering sites in the Yangtze River floodplain caused the geese to migrate 426.3 km less (Figs 1A, 1F and 2F) and terminate their migration 11 days earlier compared to the complete network (see Fig F in S1 Appendix for arrival geese at wintering sites), shifting the second infection peaks drastically, as they started earlier and grew larger.

Moreover, the infection prevalence showed one striking infection peak followed by another gentle peak in different scenarios (Fig 4A–4D), indicating that migration can temporally decrease infection prevalence. Additional simulations confirmed that migration behavior reduced the infection peak by 35% (see Fig G in S1 Appendix for infection comparison between migratory and sedentary populations), because the migration lowered direct and indirect environmental transmission while migrating, by allowing susceptible geese to escape from infection [47]. It was in line with the concept of “migratory escape” [3], i.e., the migration allows the host to “escape” from the location where pathogen infection risk is high [48]. Although these “escaped geese” were eventually infected, longer migration distance can lead to a later congregation and more recoveries, postponing the outbreaks and lowering the maximum prevalence at wintering sites. However, larger congregations and significantly shorter migration distances caused by the extreme habitat loss largely neutralize the effect of migration escape.

The migratory escape has been found in various host-pathogen relationships, such as warble fly in reindeer [3] or protozoan parasites in butterflies [49]. Although only a few studies mentioned the migratory escape for the interactions between waterfowl migration and AIVs spread and transmission [47,50,51], its effects are worth examining. For example, previous studies found that some waterfowl populations have no AIV infection during their migration, suggesting their role spreading AIV over long distances was overestimated [18,52,53]. So, what role does migratory escape play in determining the spatial and temporal patterns of AIV spread and transmission, especially in well-preserved migration network? To answer this question, we call for a more extensive sampling effort, i.e., with a larger sampling period and size [54,55] and with a broader spatial and species coverage to better capture the spatial and temporal dynamics of AIV infection at a flyway scale.

Adding age structure and varying initial population in the model slightly changed the AIV transmission patterns but did not affect the general effect of habitat loss on AIV spread. Specifically, immunological naïve geese in the population slightly increased AIV transmission (Fig H in S1 Appendix), and the smaller population size led to fewer infections (Fig I in S1 Appendix). However, the immunological naïve geese and smaller population size did not qualitatively change our conclusions about the relationship between the AIV outbreak risk, site connectivity, and number of visiting geese, or about the effects of site removal on increasing AIV outbreak risk (Fig L, J, and K in S1 Appendix).

In summary, our study explored the potential consequences of habitat loss, i.e., site removal, on spatial and temporal patterns of the AIV spread and transmission. Our simulation showed that habitat loss affects AIV distribution and prevalence. Our study emphasizes the effects of habitat loss on epidemiological dynamics in migratory waterbirds populations, and thereby on the relationships between conservation and pathogen spread. This relationship can be better understood if we combine animal tracking studies, field surveillance, and network analyzes at flyway scale in future studies.

## Methods

Our model and simulations were composed of four sections, including 1) the identification of suitable habitats, 2) migration network construction, 3) simulation of goose migration and 4) simulation of AIV transmission. First, we identified the suitable habitats for the Greater white-fronted goose by performing logistic regression analysis between the goose observation record in wetlands and environmental predictors. Second, we treated the suitable habitats as sites and used goose migration step length as maximum connection distance to construct migration network of all sites. We then removed sites from the network with intervals of 10% sites, and thus generated 6 scenarios of migration networks. Third, we used agent-based model (ABM) to simulate individual migration behavior of the Greater white-fronted goose in each network scenario. Forth, we used susceptible-infected-recovered (SIR) model to simulate the AIV transmission in the population.

### Suitable habitats identification

The distribution range of the Greater white-fronted goose in the EAAF covers a large area from 70° N in Russia to 29° N in China, which includes Mongolia, Japan, the Korean Peninsula, and the Yangtze River floodplain. We obtained the classification of breeding region, stopover region, and wintering region from the species distribution maps that generated by the Birdlife International [56]. Since stopover region and wintering region partly overlap, we classified the wintering region as between 36°N and 29°N.

All potential wetland habitats were extracted from the Global Lakes and Wetlands Database [57], and land cover maps for 1992 and 2012 were obtained from the European Space Agency CCI 300-m annual global land cover products (http://www.esa-landcover-cci.org/). Moreover, the suitability of each potential habitat was estimated by predicting the probability of waterbirds occurrence from a logistic regression model, based on the relationship between the binary observation record (i.e., presence or absence of the Greater white-fronted goose) and several environmental predictors, including water body area, elevation, longitude, and suitable foraging areas (i.e., grassland and cropland). The habitat selection followed the procedure described in a previous study [13]. We considered habitats to be suitable when the predicted probability of the goose presence exceeded 75%.

### Migration network construction

All the suitable habitats were treated as sites in the network construction. We used the coordinate of the geometric center of each site as the geographic location, and then calculated the geographic distances between all coordinate pairs. The coordinates and distances were calculated with the azimuthal equidistant projection, while the area of each site was calculated with a cylindrical equal area. Moreover, we assigned attributes, including geographic coordinates, area size and type (i.e., breeding, stopover, or wintering), to each site, and calculated the percentage of wetland habitat loss between 1992 and 2012. The suitable sites with their attributes are described in the Table B in S1 Appendix.

We only generated fall migration networks to test the effect of habitat loss, because the waterfowl migration is more likely to spread the AIV southwards [17,18]. We constructed directional links from sites with a higher latitude to sites with a lower latitude between centers of each pair, if the geographical distance *D*_*ij*_ (i.e., geographic distance between site *i* and *j*) was shorter than the migration step length *L*_*step*_ (i.e., the maximum migration distance without rest). In 50 sites (out of 98 suitable sites) area loss occurred, we generated 6 theoretical migration networks in total, by removing sites in order of the descending area of habitat loss. These theoretical networks are the complete network, and network scenarios with removal of 10%, 20%, 30%, 40%, and 50% of these sites. Each scenario had 10 sites less than the preceding scenario (See Fig B in S1 Appendix, with their corresponding basic network metrics listed in Table C in S1 Appendix).

### Simulation of goose migration

We applied a migratory flow network to simulate the geese movement over the sites [58]. Each site was assigned a variable, site attractiveness *A*_*i*_^*t*^ to represent the suitability of the site *i* at given time *t*. Each link was assigned two variables, migration resistance *R*_*ij*_ to represent the difficulty for travelling from site *i* to *j*, and the migration probability *MP*_*ij*_ to represent the likelihood for travelling from site *i* to *j*. Moreover, we assumed the attractiveness *A*_*i*_^*t*^ was negatively influenced by goose density *λ*_*i*_^*t*^, whereas the migration resistance *R*_*ij*_ was positively influenced by geographical distance *D*_*ij*_ [58]. These variables at time step *t* were calculated as:

$${MP}_{ij}=(A_{j}‐A_{i})/R_{ij}$$ (1)

$$A_{i}=-e^{k_{1}\lambda_{i}}$$ (2)

$$R_{ij}=e^{{k_{2}D}_{ij}}$$ (3)

where *k*_*1*_ and *k*_*2*_ are scaling parameters. In general, the decision was determined by goose density and distance between the sites (see supplementary method in S1 Appendix for details), and the goose prefers to select the link with greatest migration probability *MP*_*ij*_.

A total of 10,000 geese were simulated as agents in our model. Each goose was randomly assigned body mass *m*, according to a gaussian distribution at the beginning of simulation. At each time step *t*, the body mass dynamic was calculated as:

$$m^{t}=\left\{ \begin{array}{c} m^{t-1}+a,\;if\;\mathrm{resting}\\ m^{t-1}-c\times s,\;if\;\mathrm{flying} \end{array}\right.$$ (4)

where *a* is the accumulation rate during resting at a site, *c* is the body mass consumption rate during flying, *s* is the flying speed. When a resting goose cumulated enough body mass (i.e., above a threshold *φ*), the goose selected a site to migrate to in next time step.

GPS telemetry tracking revealed that Greater white-fronted goose migrates within a narrow corridor (i.e., longitude range) and makes stops for rest and refueling during fall migration [35,36]. Therefore, we setup two variables, the corridor width *w*, and the expected number of rests *n*, to constrain the sites selection. The corridor width *w* constrains the geese to migrate within a range of longitudes, and the number of rests *n* regulates the number of stopover sites before arriving at the wintering site. The detailed decision-making rules are explained in the supplementary method (Fig A in the S1 Appendix). For simplification, we did not include any goal-oriented behavior or mortality or reproduction in the model.

### Simulation of AIV transmission

We applied an SIR model to simulate the AIV transmission in the migratory population. A susceptible goose can become infected via either direct transmission, caused by direct contact between susceptible and infected geese, or indirect environmental transmission, caused by viruses in the environment. An infected goose recovered when it has been infected for a certain period *T*_*infection*_. Following previous studies [22,59], we assumed that the geese remained immune after recovery from the infection, as the antibodies to AIV can last for months in waterfowl [60].

Moreover, previous studies found that integrating frequency-dependent transmission and environmental transmission in the model best fitted the observed infection dynamics [59,61]. We therefore followed a previous framework [47], assuming that the direct transmission among geese is frequency-dependent, and the infection probability *ρ* for each susceptible goose at site *i* and time step *t* was calculated as:

$$\rho =\frac{\beta (I+E)}{N}$$ (5)

where *β* is the transmission rate parameter, *I* the number of infected geese, *N* the number of geese, *E* the amount of environmental virus at the goose scale (see below).

The amount of virus *V*^*t*^ in the environment at site *i* is calculated as:

$$V^{t}=V^{t-1}-{\eta V}^{t-1}+{\varepsilon I}^{t-1}-\eta {\varepsilon I}^{t-1}$$ (6)

where *η* is the virus decaying rate in the environment, and *ε* is the virus shedding rate. We divided the equation by shedding rate *ε* to obtain:

$$E^{t}=\frac{V^{t}}{\varepsilon }=(1-\eta )(\frac{V^{t-1}}{\varepsilon }+I^{t-1})$$ (7)

Therefore, we can use the variable *E* to represent the amount of viruses at the scale of goose, for reducing the number of variables [62]. In addition, we only modelled a single AIV strain and one goose population, to avoid the complex infection dynamics caused by cross-immune responses to multiple strains. We further assumed that the infection did not change the migration behaviors of infected geese. The simulations ended after all geese stopped migrating and no infected geese existed in the population, which allowed us to capture the full migration and complete prevalence dynamics.

### Model parameterization

A GPS telemetry tracking study suggested that Greater white-fronted goose in the EAAF acquire necessary body mass stores before starting fall migration [36], and we assume that the resting replenish the energy cost of migration. Therefore, the body mass consumption rate *c* was generally calculated as:

$$c=\frac{a{\times T}_{resting}}{D_{ns}}$$ (8)

where *T*_*resting*_ is the number of days that Greater white-fronted goose rest on stopover sites, *D*_*ns*_ is the geographic distance between the northernmost and southernmost sites. As the transmission rate parameter *β* of AIV in populations of wild goose is largely unknown [63], we used the value 0.15 (Table 1), which translates to a basic reproduction number R_0_ = 1.03 at the beginning of simulation when no virus existed in the environment, and increasing maximally to 5.97 due to the geese congregation and virus accumulation in the environment. In addition, we also conducted a sensitivity analysis for the parameter *β* by varying the value by 20% (Fig L in S1 Appendix). Other parameters were extracted from published studies (Table 1).

**Table 1:** Parameters in the agent-based model, with their abbreviation, definition, value, unit, and reference.

Parameter	Definition	Value	Unit	Reference
*N*	population size	10,0000	*goose*	[22]
*m*_*μ*_	average body mass	2075 (2075–3000)	*g*	[68]
*m*_*σ*_	standard deviation of body mass	215	*g*	[68]
φ	migration threshold of body mass	15% (12%-17.5%)	*-*	[69]
*a*	body mass accumulation rate	24.6 (24.6–30)	*g day* ^*-1*^	[69]
*c*	body mass consumption rate	0.09	*g km* ^*-1*^	-
*s*	flying speed	526 (±155)	*km day* ^*-1*^	[36]
*T*_*resting*_	resting period	17 (17–29)	*day* ^*1*^	[36]
*n*	expected number of rests	2.1 (±0.8)	*-*	[36]
*D*_*ns*_	geographic distance between the northernmost and the southernmost site in the complete network	4509	*km*	-
*L*_*step*_	step length	1710 (±476)	*km*	[36]
*ϕ*	ratio of the initial infection	0.01	*-*	-
*β*	transmission rate parameter	0.15	*day* ^*-1*^	-
*η*	virus decaying rate in environment	0.03 (0.02–0.2)	*-*	[22,59]
*T*_*infection*_	max infection period	7	*day*	[70]
*k*_*1*_	scale parameter for attractiveness	0.00001	*-*	-
*k*_*2*_	scale parameter for resistance	0.01	*-*	-
*σ*	proportion of juvenile geese	0.35	*-*	[64–66]
*ε*	Excess infection probability for juvenile geese	0.22	*-*	[67]

In addition, we simulated AIV transmission within a sedentary population for illustrating the effects of migration. We analyzed the influence of a difference in age structure (i.e., juvenile/adult ratio) by dividing the population into juvenile and adult groups and setting the juvenile group had higher infection probability (Table 1). According to previous studies, the proportion of juvenile geese was set as 35% in the migratory population [64–66], and the juvenile geese have a 22% greater risk to be infected, compared to the adult geese [67]. We also analyzed the influence of a declining population size with habitat loss, by estimating the population size in each scenario. We estimated the population sizes by implementing a modified regression relationship between ratio of population change and ratio of functional connectivity change to estimate the population sizes, in line with a previous study [30]. The description of the regression relationship and the estimated functional connectivity and corresponded population sizes are summarized in Table D in S1 Appendix.

### Model analysis

In each network scenario, we initiated the model with all geese at the northernmost site, with an initial infection prevalence 1%. No virus pre-existed in environment at any site at the beginning of the simulations. In the simulation, one time step was equivalent to one day.

To investigate the infection dynamics during migration, we counted the number of infected geese via direct transmission and indirect environmental transmission. We also calculated the effective reproduction number *R*_*0*_ (i.e., the sum of the average number of new infections) for each site at each time step as below, *R*_*0*_>1 indicates an outbreak [22]:

$$R_{0}=\beta T\frac{S}{N}+\beta \frac{1}{\eta }\frac{S}{N}$$ (9)

For each network, the simulation iterated 1,000 times and all outputs were averaged. For each iteration, we exported the selected link of each goose at each time step, which were used to generate the visited migration network for performing further analysis. Moreover, we used the average link repetitions (i.e., over 1000 iterations) as weight score for calculating the weighted in-degree of each site to represent both the connection with other sites and the number of geese arriving on the focal site for resting. We also calculated the average travelling distance of the population and the number of sites, the number of links, and the link densities as network-level metrics to study the effects of site removal on network connectivity [71].

### Statistical analysis

We performed a generalized linear model (GLM) to examine whether the habitat loss can increase the outbreak risk in remaining sites. We selected the maximum *R*_*0*_ across time steps at each site to represent the outbreak risk. The independent predictors were the weighed in-degree and network scenario. Data generated from all six network scenarios were integrated to perform the GLM analysis. We used the Kruskal-Wallis test to examine the difference in basic metrics of the visited network in different scenarios, and the t-test to examine the difference in the basic reproduction number R_0_ at each site in the different scenarios. In this study, the environmental factors were extracted in ArcMap 10.2.1, the agent-based model was constructed in Netlogo 6.1.1 (S2 Appendix), the background map of the East Asian was extracted from package *“maps”* in R environment [72], the numerical data used in figures are included in S3 Data. All the data processing and statistical analysis were performed in R 4.0.5.

## Supporting information

S1 Appendix Supplementary method, figures and tables (PDF). (PDF)

S2 Appendix The agent-based model for simulating geese migration and AIV transmission (Netlogo). (ZIP)

S1 Data The numerical data used in figures (Rdata). (ZIP)
