# Supplementary material for: Habitat loss exacerbates pathogen spread: An Agent-based model of avian influenza infection in migratory waterfowl
Source: PLoS Comput Biol. 2022 Aug 18;18(8):e1009577. doi: 10.1371/journal.pcbi.1009577 (PMC9426877; doi:10.1371/journal.pcbi.1009577)
Supplement: S1 Appendix — (PDF) [file pcbi.1009577.s001.pdf]

Habitat loss exacerbates pathogen spread: An Agent-based model of avian influenza infection in migratory waterfowl

Shenglai Yin[1,3], Yanjie Xu[2,3], Mingshuai Xu[1], Mart C.M. de Jong[4], Mees R.S. Huisman[3], Andrea Contina[5], Herbert H. T. Prins[6], Zheng Y. X. Huang[1*], Willem F. de Boer[3]

[1] College of Life Science, Nanjing Normal University, Nanjing, China

[2] The Finnish Museum of Natural History, University of Helsinki, Helsinki, Finland

[3] Wildlife Ecology and Conservation Group, Wageningen University, Wageningen, The Netherlands

[4] Quantitative Veterinary Epidemiology Group, Wageningen University, Wageningen, The Netherlands

[5] ~~Department of Microbiology and Plant Biology, Center for Earth Observation and Modeling, University of Oklahoma, Norman, Oklahoma, USA,~~

[6] Department of Animal Sciences, Wageningen University, Wageningen, The Netherlands

[*]Corresponding author:

Zheng Y.X. Huang

Email: zhengyxhuang@gmail.com (ZXYH)

Supplementary method

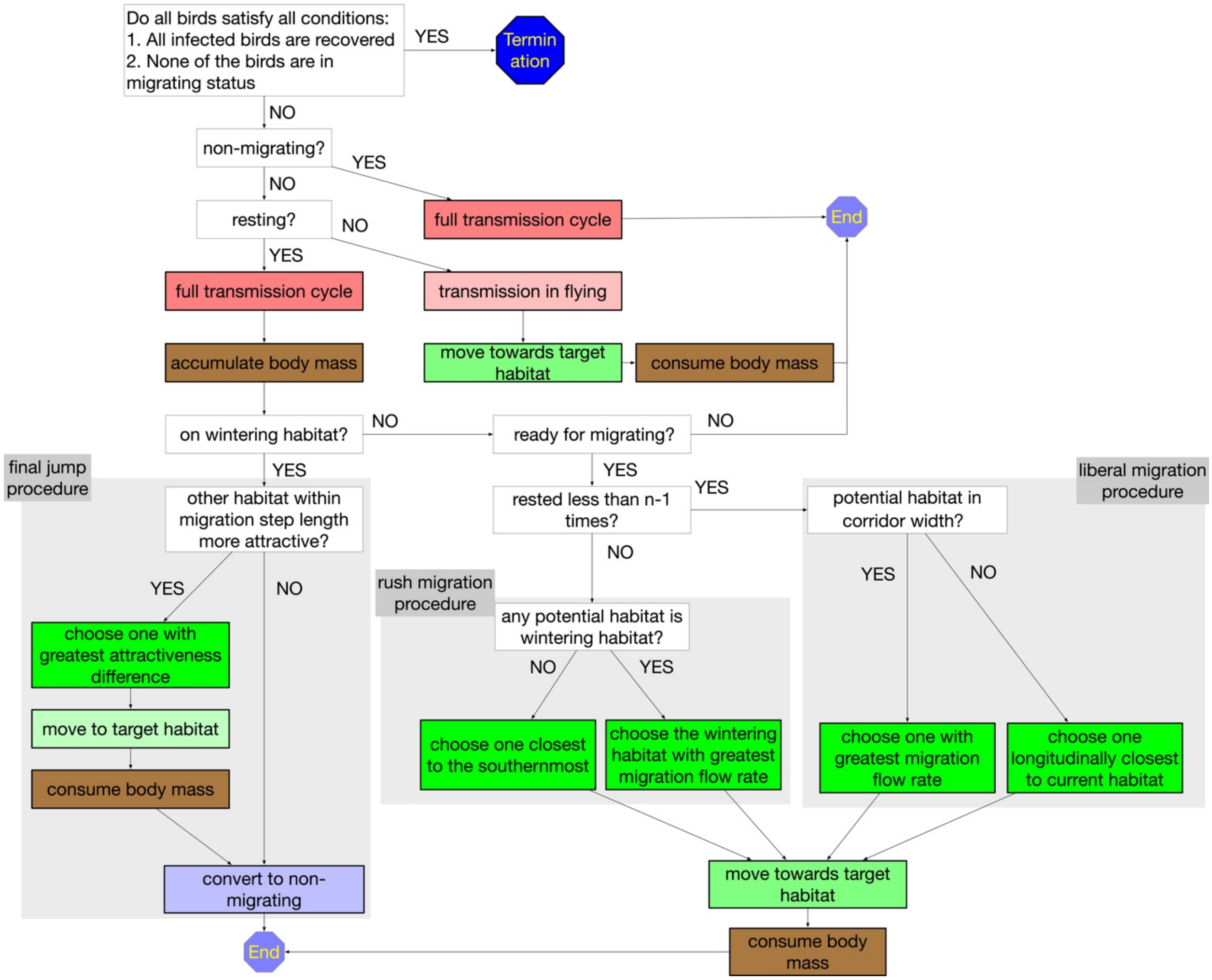

Fig A. Description of the decision-making rules for simulated geese migration. White boxes represent YES/NO judging procedures; colored boxes represent action procedures; grey boxes are decision-making procedures regarding *liberal migration*, *rush migration* and *final jump*.

At the beginning of the simulation, 10,000 migratory Greater white-fronted geese *Anser albifrons* were created, and each one was assigned a binomial variable, the non-migrating status (YES/NO), and various numeric variables, including the expected number of rests (n), flying speed (s), body mass (m), body mass accumulation rate (a), and body mass consumption rate (c), etc. The process of each goose's decision making is described below.

In each simulation time step, the model firstly examines whether all geese have recovered from the infection, and whether all geese are resting on their wintering grounds. If the two

conditions are satisfied, the whole simulation terminates. By satisfying these two conditions to terminate the simulation, we capture the whole infection development and migration process with the same model iteration. If any one of the conditions is not satisfied, the following procedures was executed.

First, the *non-migrating?* status, which is the switch of moving among sites, of each goose is examined. The geese in *non-migrating* status (YES) are those which either have finished their migration, or have arrived at a site which has no links coming out of it. Since these geese do not migrate anymore, they can only execute the *full transmission cycle* procedure. For a goose that is not in the *non-migrating* status (NO), it checks whether it is *resting* in a site. If not, it executes the *transmission in flying* procedure to accumulate their infection period, and to *move toward their target site* and *consumes body mass* accordingly. If yes, the goose executes the *full migration cycle* procedure and *accumulates body mass*.

For the goose resting in a site, it continuously examines whether it is resting *in a wintering site*. If yes, it executes the *final jump procedure* to finish the last step for the migration. Whereas for a goose not resting *in a wintering site*, it makes action decisions based on its body mass condition and the times it has already rested. If the body mass does not reach the migration threshold, i.e., $m \times (1+\varphi)$, the goose has not yet prepared for the next migration step, thereby ending its activities in this time step. If the body mass reaches the threshold, it checks whether itself *rested less than n-1 times* so far. If yes, it means the goose can still rest more times during the migration, thereby it executes the *liberal migration procedure*; if not, the goose needs to arrive at one of the wintering sites as soon as possible, thereby it executes the *rush migration procedure*.

The liberal migration procedure

Satellite tracking studies [1,2] revealed that Greater white-fronted goose migrate within a narrow corridor (i.e., longitude range), thus a goose selects its target site as the one which satisfies two conditions: 1) the site is located within the corridor width (w); and 2) it has the greatest migration flow rate (MF_{ij}) among all the potential sites. If there are no potential sites located within the corridor width (w), the goose selects one that is longitudinally closest to its currently resting sites. This ensures the goose migrates within the corridor, or at least does not deviate too far from the main corridor.

The rush migration procedure

A goose executing the rush migration procedure is not restricted by the migration corridor width (w). It preferably selects the overwintering site with the greatest migration flow rate (MF_{ij}). If none of the overwintering sites are connected with the current resting site, the goose selects one stopover site that is closest to the southernmost site. This ensures the goose accomplishes its migration with minimal extra expected number of resting (n).

When a goose, either in the *liberal migration procedure* or in the *rush migration procedure*, has selected its target site, it *moves towards the target site* and *consumes body mass* accordingly. After the above procedures, its decision-making procedure ends for this time step.

The final jump procedure

To avoid that all geese concentrate in a only a few wintering sites, a goose executes the *final jump procedure* after it first arrives at one of the wintering site. If there are other wintering sites connected to its current one, it selects the site with the greatest attractiveness, *moves to the target site* directly, and *consumes body mass* accordingly. Then the goose converts to a *non-migrating* status and ends its decision-making procedure in this time step. If there is no other wintering site with greater attractiveness, or no other wintering site connects to the current one, the goose converts to a *non-migrating* status and ends its decision-making in this time step.

Supplementary figures

Fig B The theoretical fall migration networks of Greater white-fronted goose in the EAAF. A) the complete network, and B-F) the network scenarios with increasing site removal. The dots are the suitable sites selected by logistic regression procedure, the colours represent the order of site removal, and the numbers are site IDs. The yellow and blue shadows depict the breeding and wintering regions. The base layer maps were generated in R environment using the "*maps*" package (Original S code by Richard A. Becker, Allan R. Wilks. R version by Ray Brownrigg. Enhancements by Thomas P Minka and Alex Deckmyn. (2016). maps: Draw Geographical Maps. R package version 3.1.1. http://CRAN.R-project.org/package=maps)[3].

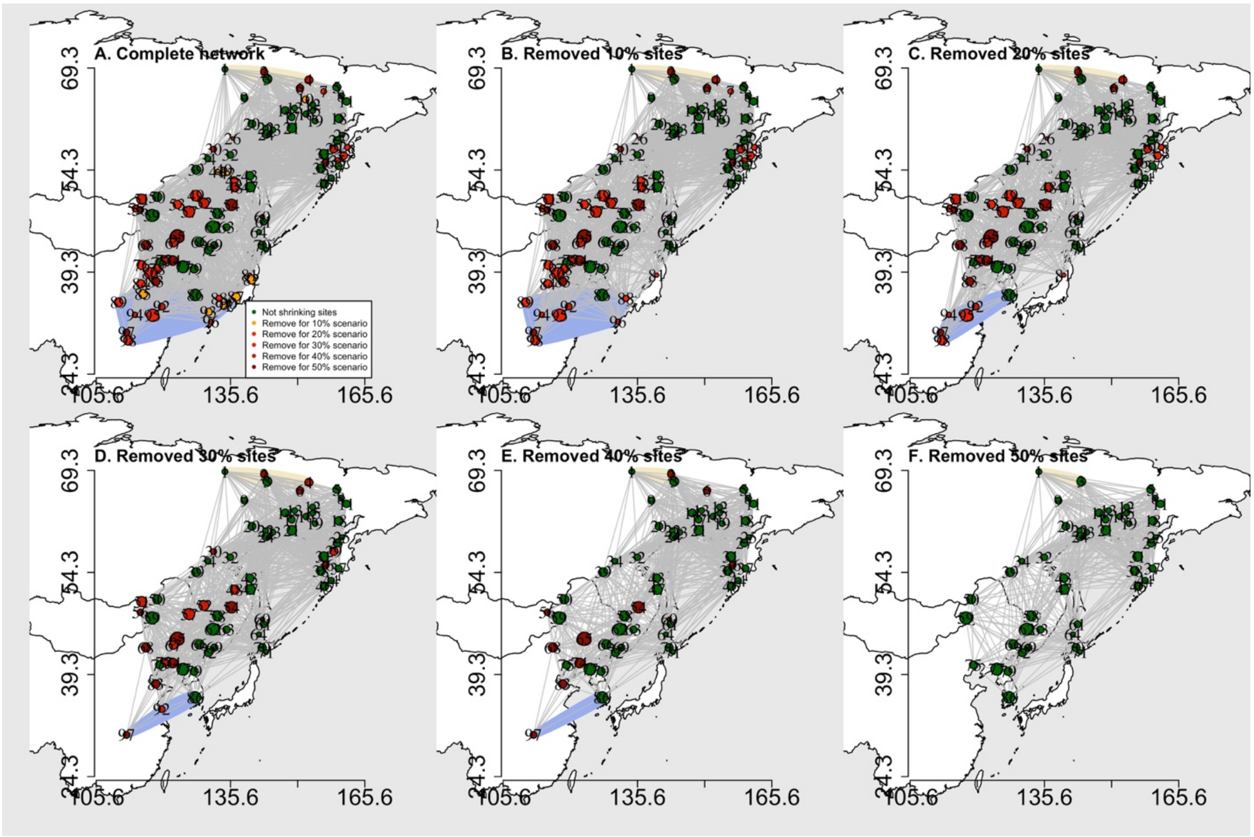

Fig C The number of geese on each site each day. The yellow and blue shadows are the breeding and wintering range. A) the complete network, and B-F) the network scenarios of 10%, 20%, 30%, 40% and 50% site removal.

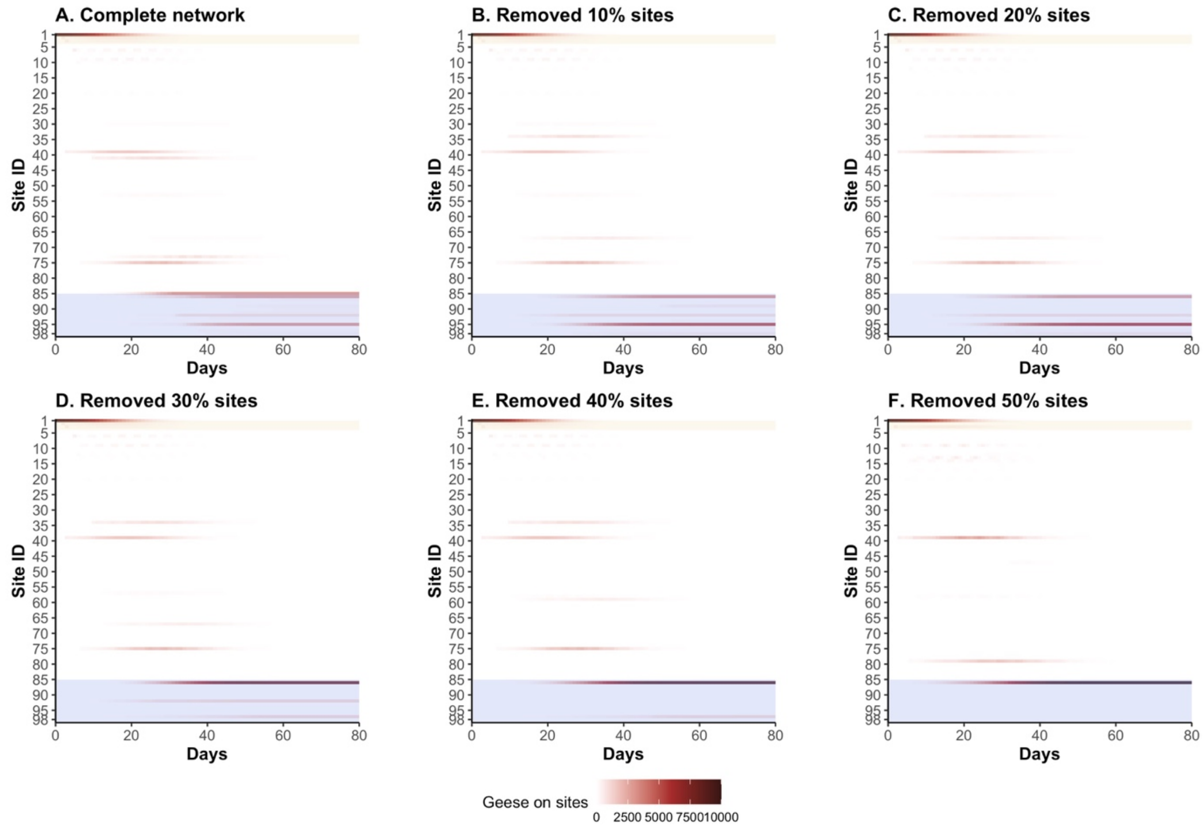

Fig D The effects of increasing sites removal A) and B), and weighted in-degree C) and D) on the basic reproduction number R0 at each site. In A) and B), x-axis labels are the scenario of the complete network, and network scenarios of 10%, 20%, 30%, 40% and 50% removal of sites; in C) and D), black line represents the GLM fit, and grey shaded area represent the 95% confidence interval. The asterisk represents the levels of statistical difference (** for p<0.01), compared to the complete network scenario. Coloured dots are the maximum R_0 values generated by agent-based model simulations under different network scenarios. A) and C) were generated with simulation outputs at breeding and stopover sites; B) and D) were generated with simulation outputs at wintering sites.

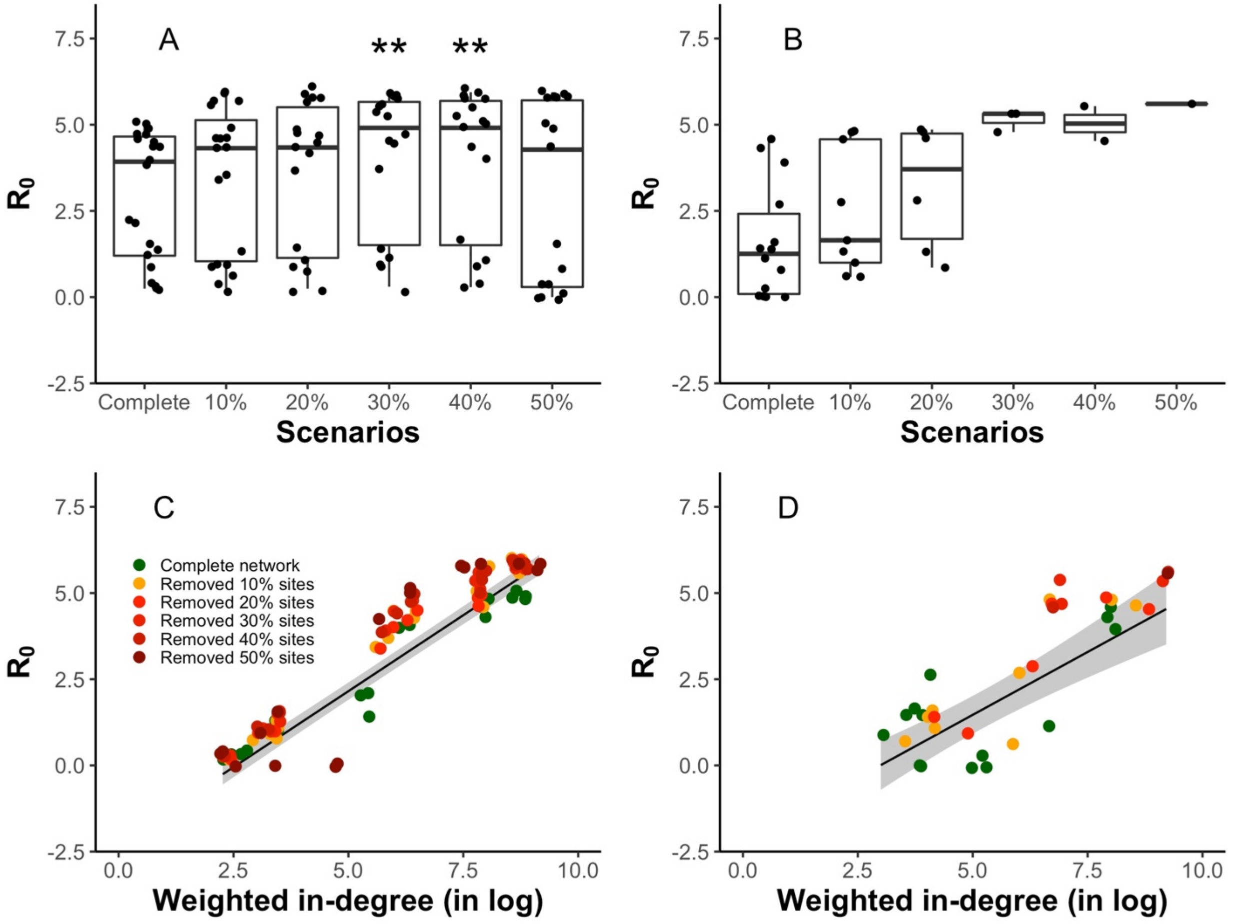

Fig E Averaged virus cumulation across all visited sites. Line colours represent the virus accumulation under different network scenarios.

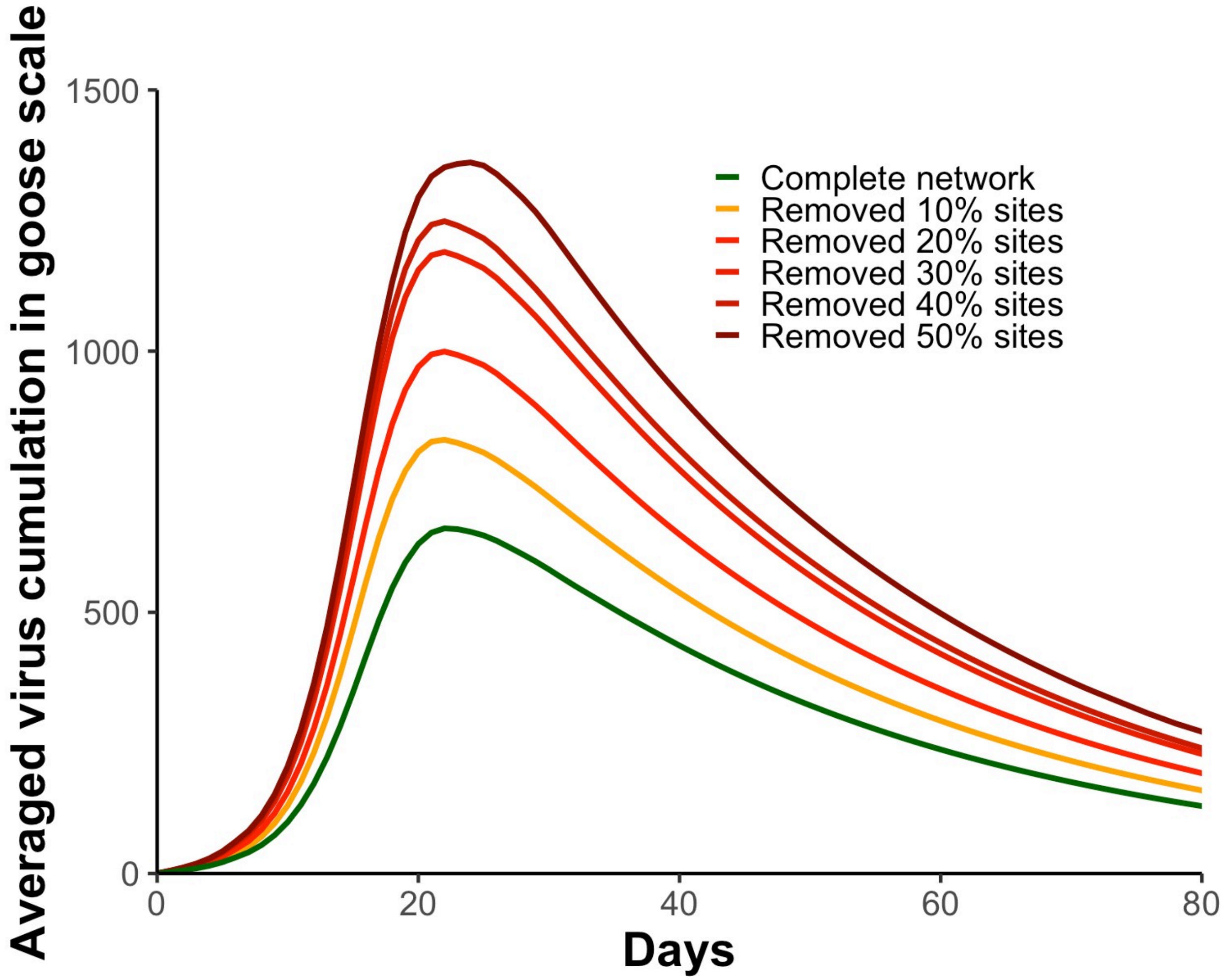

Fig F The number of geese at wintering sites over time. The dashed lines mark the time when all geese arrived at the wintering sites in the scenario of complete network (green) and the scenario of removed 50% of sites (orange).

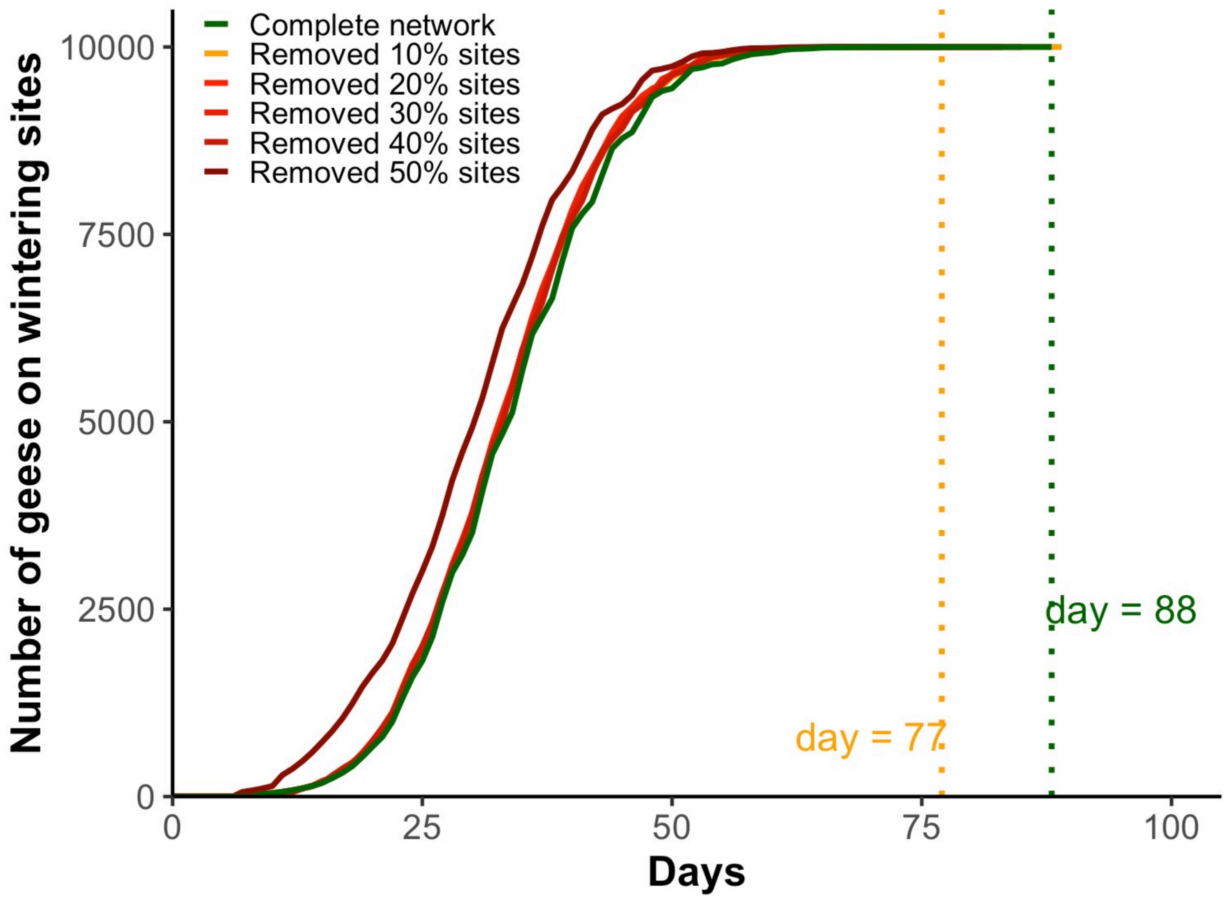

Fig G Comparison of the infection prevalence between sedentary population (blue) and migratory population (green) in complete network. A) number of infections; B) number of cumulative infections; C) number of infections caused by direct transmission; D) number of infections caused by indirect environmental transmission.

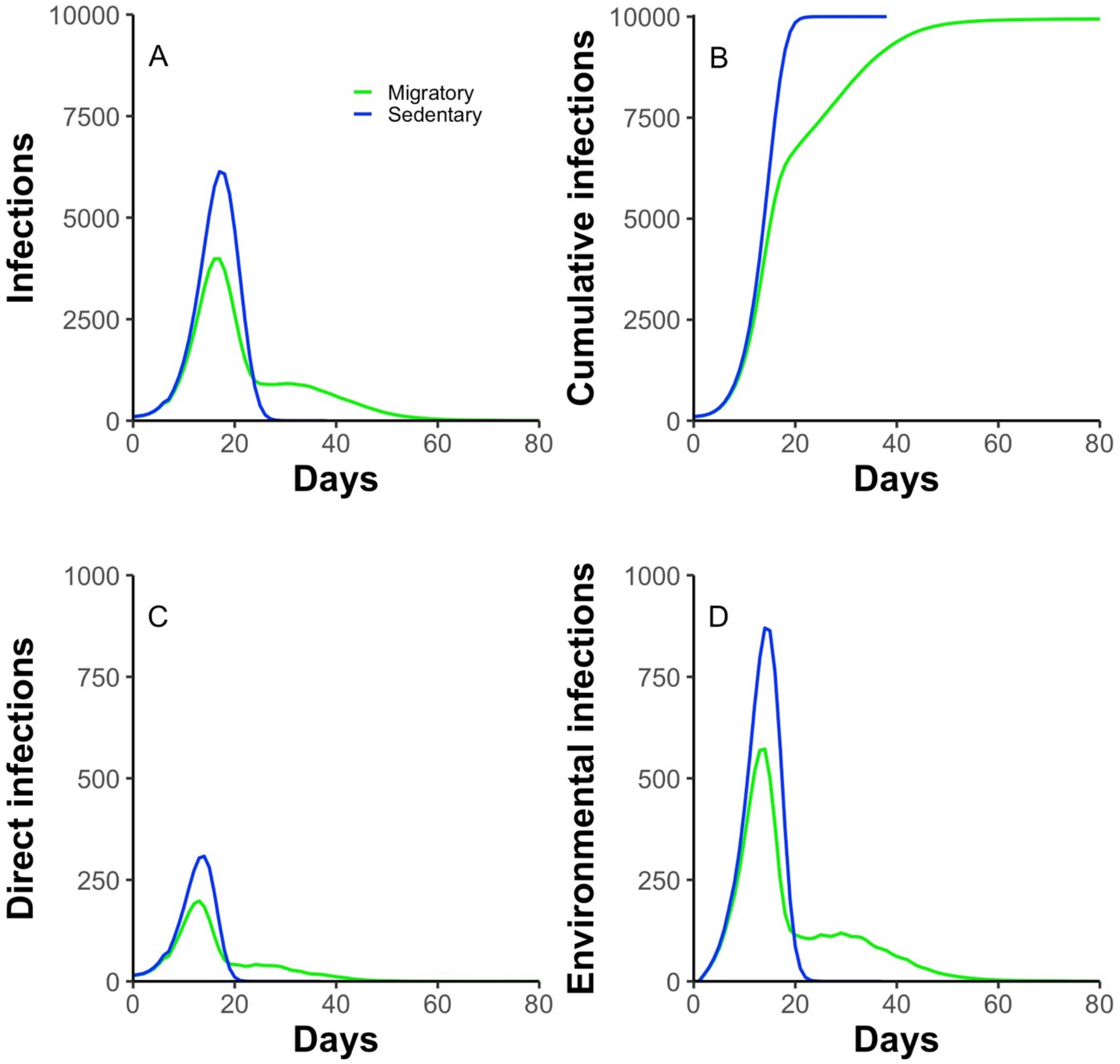

Fig H Infection dynamics generated in the migratory population with age structure. A) number of infections; B) number of infections at the wintering sites; C) number of infections caused by direct transmission; D) number of infections caused by indirect environmental transmission. Line colours represent the infection dynamics under different network scenarios. The proportion of juvenile goose was 0.35 [4–6], and the juvenile goose had a 22% greater probability for infection [7].

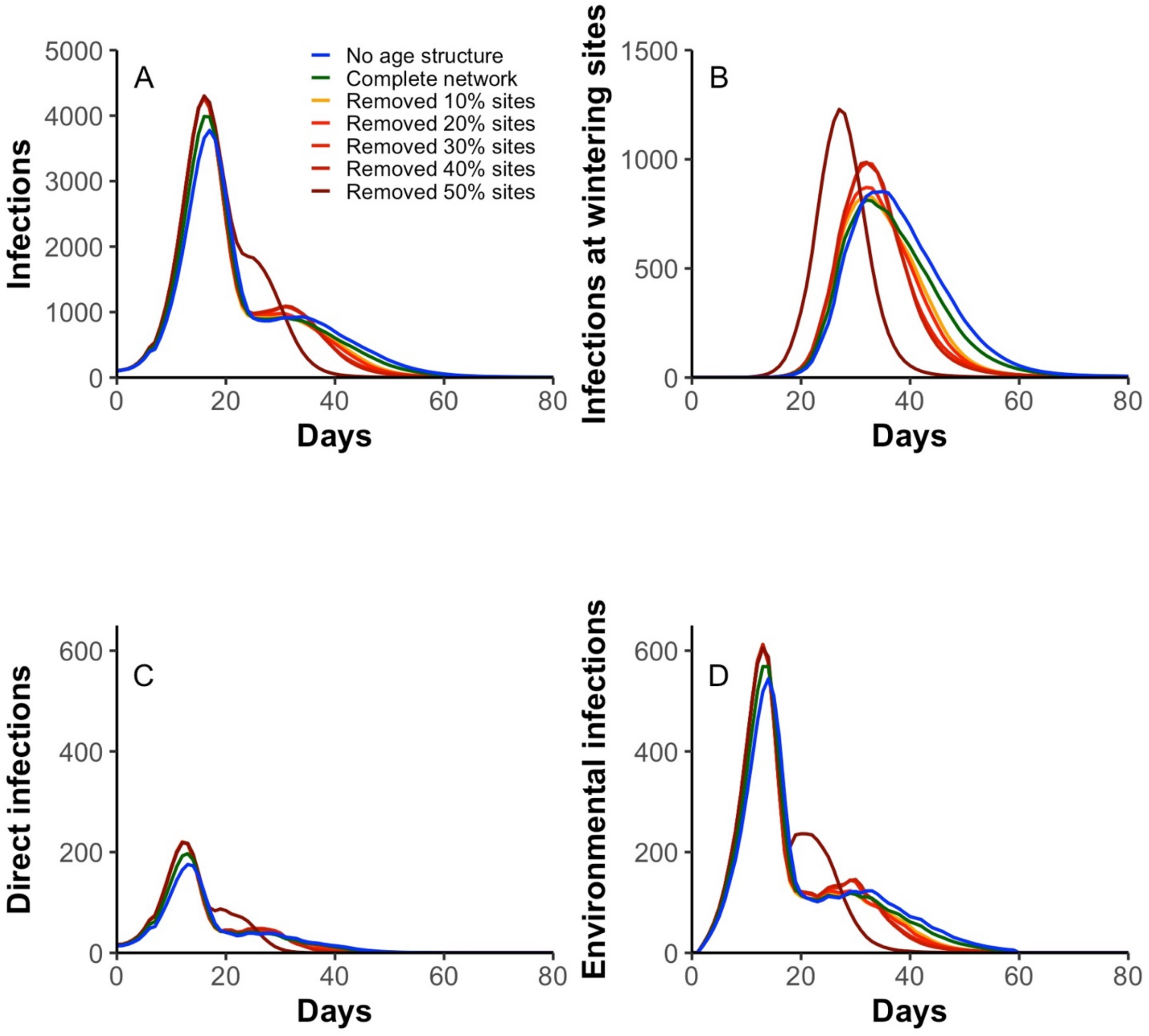

Fig I Infection dynamics generated in the migratory population with declining population size. A) number of infections; B) number of infections at the wintering sites; C) number of infections caused by direct transmission; D) number of infections caused by indirect environmental transmission. Line colours represent the infection dynamics under different network scenarios. The population sizes were generated by following the previously reported regression equation between function connectivity of migration network and population size [8] (see Table D for the estimations of function connectivity and population sizes).

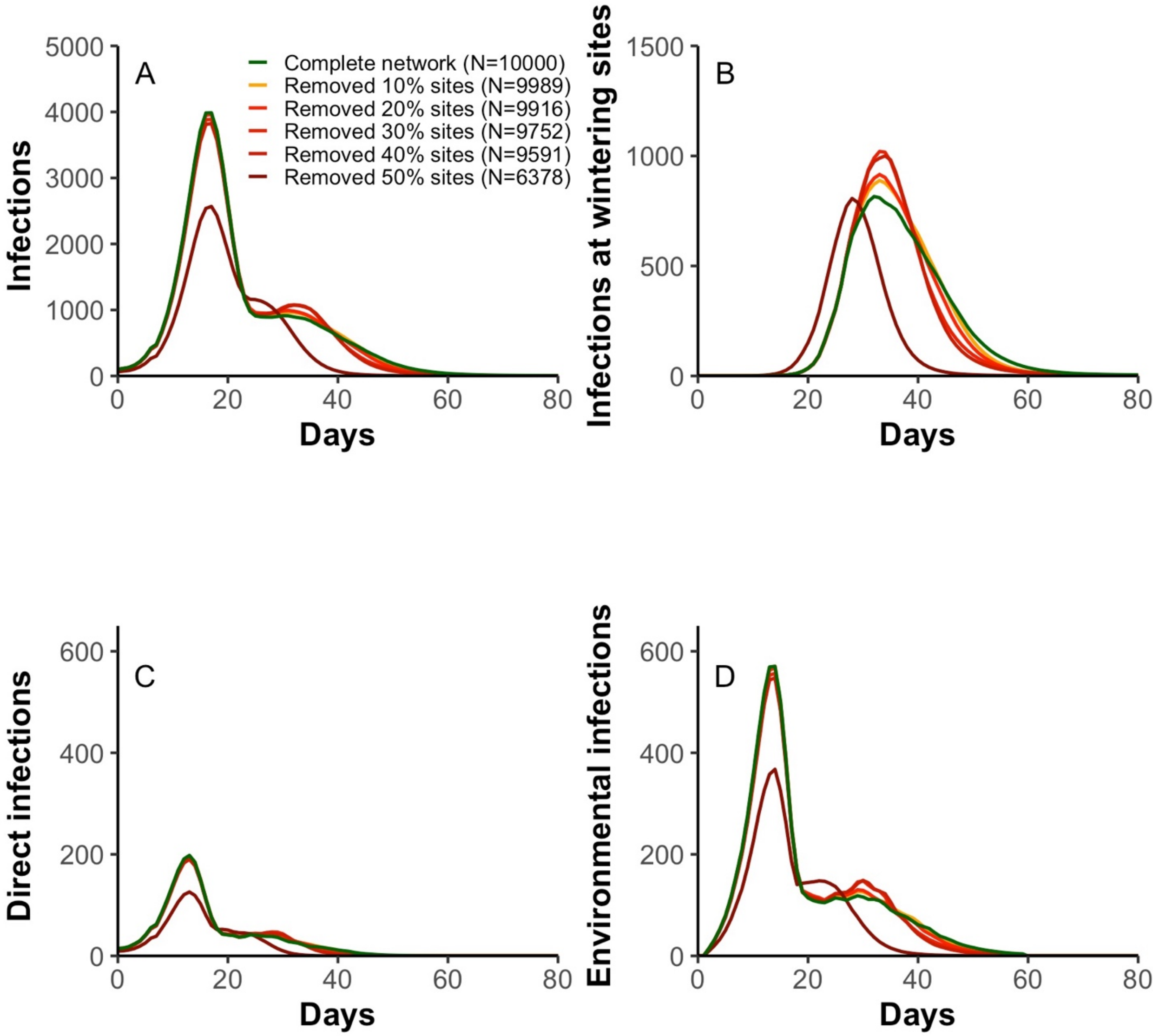

Fig J The effects of A) increasing sites removal and B) weighted in-degree on the basic reproduction number R_0 at each site for population with age structure. A) x-axis labels are the scenario of the complete network, and network scenarios of 10%, 20%, 30%, 40% and 50% removal of sites; B) black line represents the GLM fit, and grey shaded area represent the 95% confidence interval. The asterisk represents the levels of statistical difference (* for p<0.05, and *** for p<0.001), compared to the complete network scenario. Coloured dots are the maximum R_0 values generated by agent-based model simulations under different network scenarios.

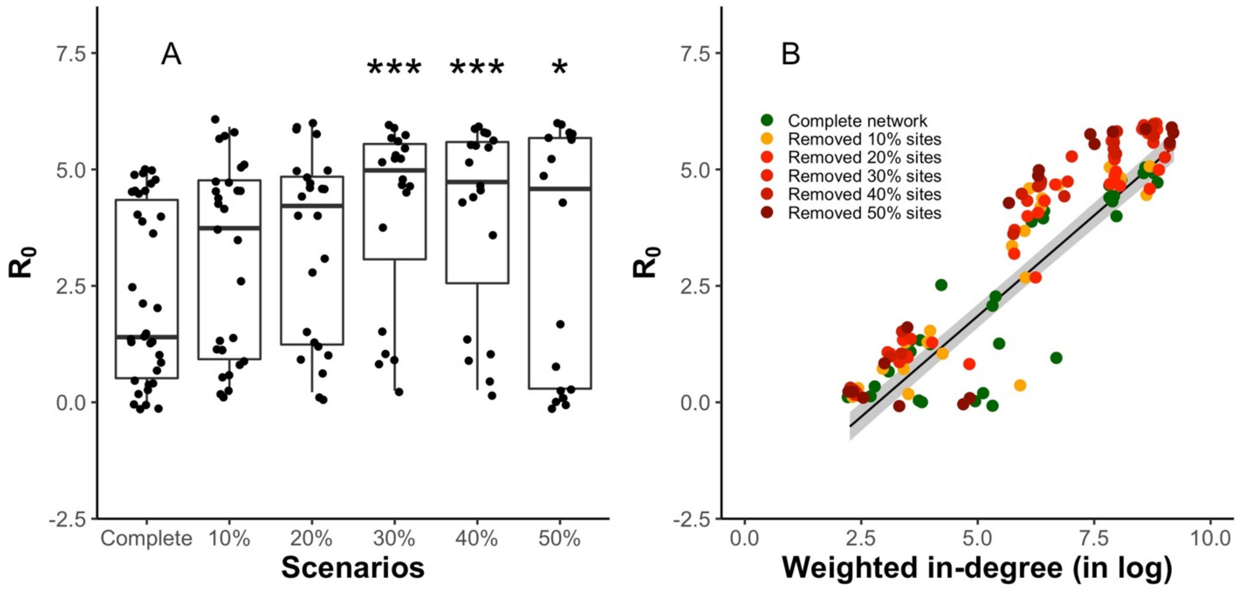

Fig K The effects of A) increasing sites removal and B) weighted in-degree on the basic reproduction number R_0 at each site for population with declining population size. A) x-axis labels are the scenario of the complete network, and network scenarios of 10%, 20%, 30%, 40% and 50% removal of sites; B) black line represents the GLM fit, and grey shaded area represent the 95% confidence interval. The asterisk represents the levels of statistical difference (** for p<0.01, and *** for p<0.001), compared to the complete network scenario. Coloured dots are the maximum R_0 values generated by agent-based model simulations under different network scenarios.

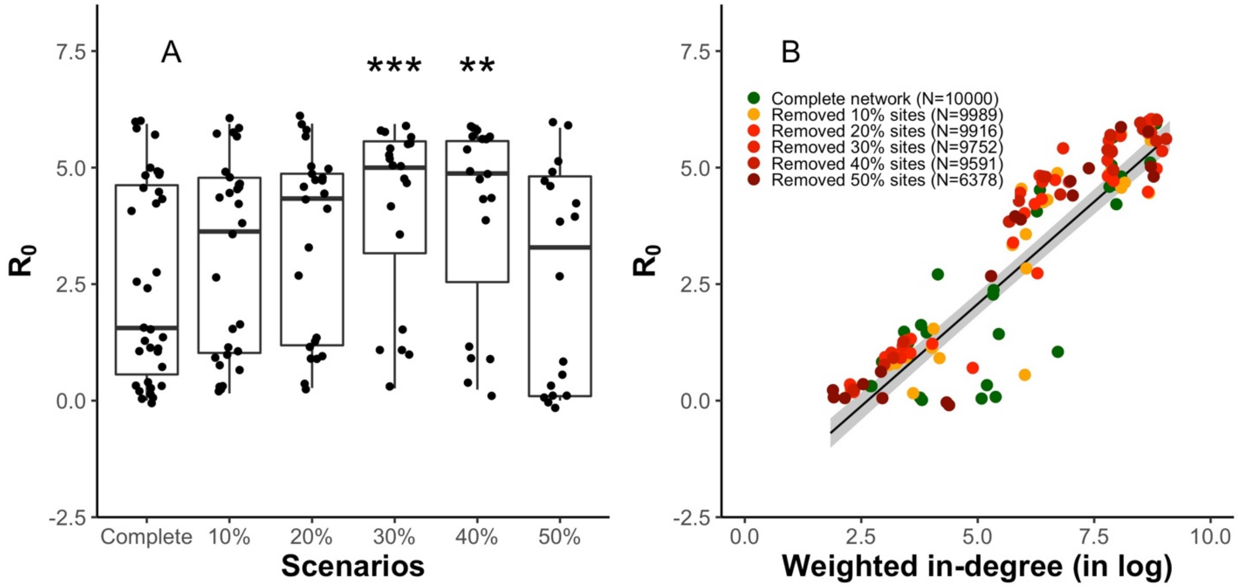

Fig L Infection dynamics generated from the sensitivity analyses by altering the transmission rate parameter β with 20% intervals. Line colours represent the infection dynamics under different β value.

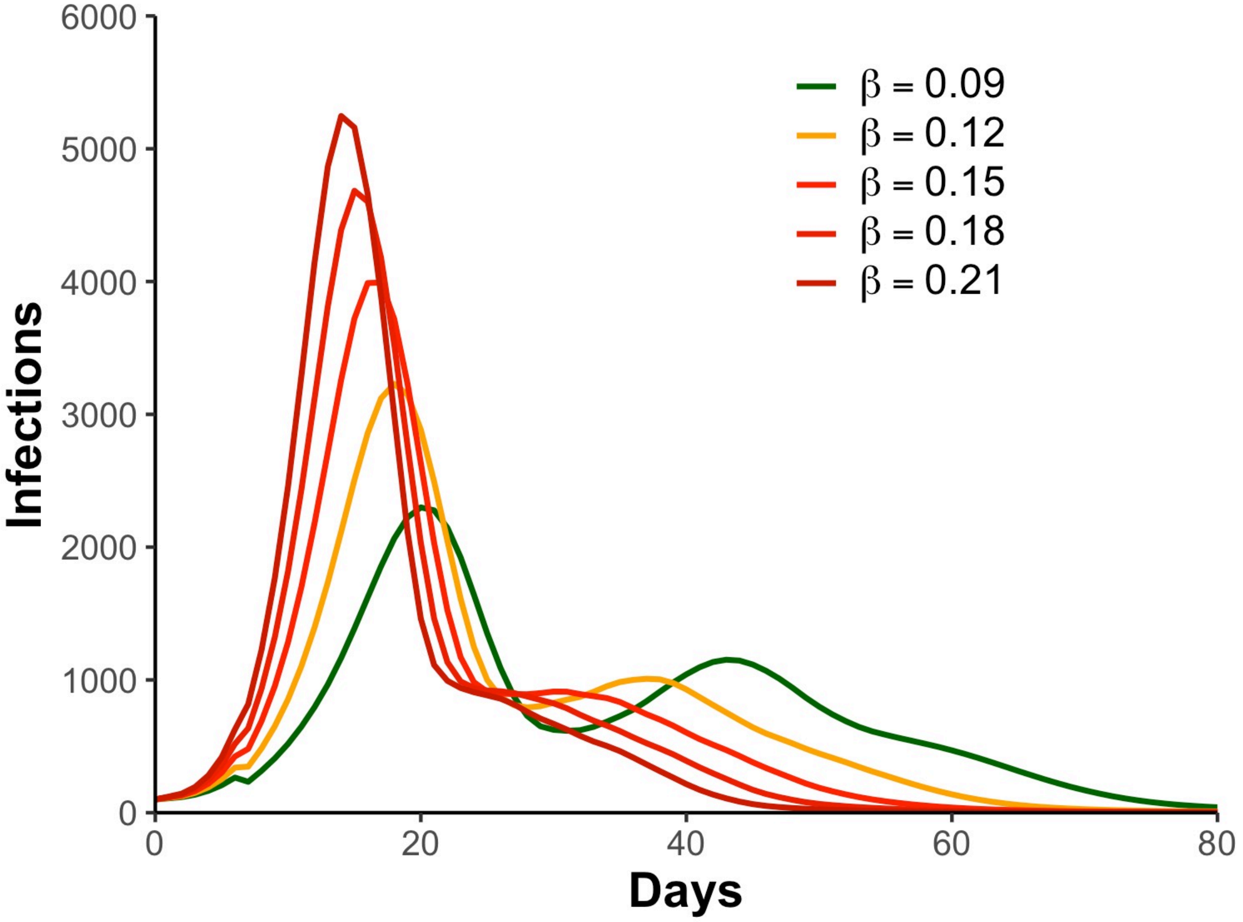

Table A The visited sites in each scenario, accompanied with the number of visiting geese and R_0. The R_0 is the maximum R_0 through simulation; the birds are the number of geese that visited the site; the #N/As indicate the sites were removed in the corresponded scenario. Bold records indicate the sites where R_0 drastically increased (>1).

Site ID	Complete Network		Removed 10% sites		Removed 20% sites		Removed 30% sites		Removed 40% sites		Removed 50% sites	
	R_0	Birds	R_0	Birds	R_0	Birds	R_0	Birds	R_0	Birds	R_0	Birds
1	5.57	10000	5.97	10000	5.97	10000	5.97	10000	5.97	10000	5.97	10000
2	5.53	501	5.92	501	5.91	500	5.91	501	5.92	502	#N/A	0
3	5.43	412	5.82	413	5.81	411	5.81	413	5.82	413	5.92	501
4	0.00	0	0.00	0	0.00	0	0.00	0	#N/A	0	#N/A	0
5	0.00	0	0.00	0	0.00	0	0.00	0	0.00	0	0.00	0
6	5.34	412	5.75	413	5.70	411	5.69	413	5.73	413	#N/A	0
7	2.30	43	5.00	99	#N/A	0	#N/A	0	#N/A	0	#N/A	0
8	0.38	2	1.10	6	0.00	0	0.00	0	0.00	0	0.00	0
9	5.05	250	5.34	250	5.33	249	5.35	249	5.33	249	5.83	464
10	4.36	98	#N/A	0	#N/A	0	#N/A	0	#N/A	0	#N/A	0
11	0.00	0	0.00	0	0.00	0	0.00	0	0.00	0	0.00	0
**12**	**1.33**	**7**	**3.74**	**59**	**4.44**	**97**	**4.46**	**98**	**4.41**	**98**	**0.00**	**88**
**13**	**0.00**	**0**	**0.00**	**0**	**3.86**	**59**	**3.89**	**59**	**3.89**	**60**	**5.65**	**481**
**14**	**0.00**	**0**	**0.00**	**0**	**0.98**	**4**	**1.00**	**4**	**1.06**	**4**	**5.71**	**249**
15	0.00	0	0.92	4	1.01	7	0.98	6	1.01	7	0.00	18
16	0.00	0	0.00	0	0.00	0	0.00	0	0.01	0	0.04	4
**17**	**1.10**	**7**	**0.00**	**0**	**0.00**	**0**	**0.00**	**0**	**0.00**	**0**	**4.28**	**55**
18	0.00	0	0.00	0	0.00	0	0.00	0	0.00	0	0.00	0
19	0.00	0	0.00	0	0.00	0	0.00	0	0.00	0	0.00	0
20	4.67	59	4.92	59	4.90	59	4.91	59	4.91	59	5.10	80
21	0.00	0	0.00	0	0.00	0	0.00	0	0.00	0	0.98	4
22	1.44	4	1.52	4	1.60	4	1.51	4	1.51	4	1.54	5
23	0.00	0	0.00	0	0.01	0	0.00	0	0.00	0	0.01	0
24	0.28	2	0.28	2	0.28	2	0.30	2	0.29	2	0.29	2
25	0.00	0	0.00	0	0.00	0	0.00	0	0.00	0	0.00	0
26	0.00	0	0.00	0	0.00	0	#N/A	0	#N/A	0	#N/A	0
27	0.00	0	0.00	0	0.00	0	0.00	0	0.00	0	0.00	0
28	0.00	0	0.00	0	0.00	0	#N/A	0	#N/A	0	#N/A	0
29	0.00	0	0.00	0	0.00	0	0.00	0	#N/A	0	#N/A	0
30	2.36	79	4.01	106	0.00	0	0.00	0	#N/A	0	#N/A	0
31	0.00	0	0.00	0	0.00	0	0.00	0	0.00	0	0.00	0

32	0.28	6	0.81	8	0.00	0	0.00	0	0.00	0	0.00	0
33	0.00	0	0.00	0	0.00	0	#N/A	0	#N/A	0	#N/A	0
**34**	**0.00**	**0**	**5.51**	**850**	**5.54**	**850**	**5.55**	**852**	**5.57**	**850**	**0.00**	**0**
35	0.00	0	0.00	0	#N/A	0	#N/A	0	#N/A	0	#N/A	0
36	0.00	0	0.00	0	0.00	0	0.00	0	0.00	0	#N/A	0
37	0.00	0	0.00	0	0.00	0	0.00	0	0.00	0	0.00	0
38	0.00	0	0.00	0	0.00	0	0.00	0	0.00	0	0.00	0
39	5.53	1517	5.93	1515	5.94	1576	5.94	1578	5.94	1579	5.94	2515
40	3.36	91	#N/A	0	#N/A	0	#N/A	0	#N/A	0	#N/A	0
41	5.17	880	#N/A	0	#N/A	0	#N/A	0	#N/A	0	#N/A	0
42	0.17	2	#N/A	0	#N/A	0	#N/A	0	#N/A	0	#N/A	0
43	0.00	0	0.00	0	0.00	0	0.00	0	0.00	0	0.00	0
44	0.00	0	0.00	0	0.00	0	0.00	0	0.00	0	0.00	0
45	0.00	0	0.00	0	#N/A	0	#N/A	0	#N/A	0	#N/A	0
46	0.00	0	0.00	0	0.00	0	0.00	0	0.00	0	0.00	0
47	0.00	0	0.00	0	0.00	0	0.00	0	0.00	0	0.01	89
48	0.00	0	0.00	0	0.00	0	0.00	0	#N/A	0	#N/A	0
49	1.12	6	1.17	6	1.15	6	#N/A	0	#N/A	0	#N/A	0
50	0.00	0	0.00	0	0.00	0	0.00	0	0.00	0	0.00	0
51	0.00	0	0.00	0	0.00	0	0.00	0	#N/A	0	#N/A	0
**52**	**0.00**	**0**	**0.00**	**0**	**0.00**	**0**	**1.21**	**6**	**#N/A**	**0**	**#N/A**	**0**
53	4.43	147	4.68	146	4.74	147	#N/A	0	#N/A	0	#N/A	0
54	0.00	0	0.00	0	0.00	0	0.00	0	0.00	0	#N/A	0
55	0.00	0	0.00	0	0.00	0	#N/A	0	#N/A	0	#N/A	0
56	0.00	0	0.00	0	0.00	0	0.00	0	0.00	0	#N/A	0
**57**	**0.27**	**2**	**0.28**	**2**	**0.27**	**2**	**4.73**	**148**	**#N/A**	**0**	**#N/A**	**0**
**58**	**0.00**	**0**	**0.00**	**0**	**0.00**	**0**	**0.00**	**0**	**4.78**	**114**	**4.94**	**108**
**59**	**0.00**	**0**	**0.00**	**0**	**0.00**	**0**	**0.00**	**0**	**5.07**	**441**	**0.00**	**0**
60	0.00	0	0.00	0	0.00	0	0.00	0	0.00	0	#N/A	0
61	0.00	0	0.00	0	0.00	0	0.00	0	0.00	0	0.00	0
62	0.00	0	0.00	0	0.00	0	0.00	0	0.29	2	0.38	2
63	0.00	0	0.00	0	0.00	0	0.00	0	0.00	0	0.00	0
64	0.00	0	0.00	0	0.00	0	0.00	0	0.00	0	0.00	0
65	0.00	0	0.00	0	0.00	0	0.00	0	0.00	0	#N/A	0
66	0.00	0	0.00	0	0.00	0	0.00	0	0.00	0	0.00	0
**67**	**1.59**	**85**	**5.17**	**534**	**5.12**	**441**	**5.17**	**440**	**#N/A**	**0**	**#N/A**	**0**
68	0.00	0	0.00	0	0.00	0	0.00	0	0.00	0	#N/A	0
69	0.00	0	0.00	0	0.00	0	0.00	0	0.00	0	0.00	0
70	0.00	0	0.00	0	0.00	0	0.00	0	0.00	0	0.00	0
71	0.00	0	0.00	0	0.00	0	0.00	0	0.00	0	0.00	0

72	0.00	0	0.00	0	0.00	0	0.00	0	0.00	0	0.00	0
73	4.83	766	0.00	0	0.00	0	0.00	0	#N/A	0	#N/A	0
74	0.00	0	0.00	0	0.00	0	0.00	0	0.00	0	#N/A	0
75	5.30	1777	5.65	1775	5.68	1869	5.66	1869	5.69	1870	0.00	0
76	0.00	0	0.00	0	#N/A	0	#N/A	0	#N/A	0	#N/A	0
77	0.00	0	0.00	0	0.00	0	0.00	0	0.00	0	0.00	0
78	0.00	0	0.00	0	#N/A	0	#N/A	0	#N/A	0	#N/A	0
79	0.21	3	0.25	3	0.27	3	0.00	0	0.00	0	5.75	1547
80	0.00	0	0.00	0	#N/A	0	#N/A	0	#N/A	0	#N/A	0
81	0.00	0	0.00	0	0.00	0	#N/A	0	#N/A	0	#N/A	0
82	0.00	0	#N/A	0	#N/A	0	#N/A	0	#N/A	0	#N/A	0
83	0.00	0	0.00	0	0.00	0	0.00	0	0.00	0	#N/A	0
84	0.00	0	0.00	0	#N/A	0	#N/A	0	#N/A	0	#N/A	0
85	5.09	3162	#N/A	0	#N/A	0	#N/A	0	#N/A	0	#N/A	0
86	4.80	2678	5.35	2989	5.40	2727	5.32	8091	5.54	9074	5.60	10000
87	0.03	147	#N/A	0	#N/A	0	#N/A	0	#N/A	0	#N/A	0
88	1.54	33	0.67	42	#N/A	0	#N/A	0	#N/A	0	#N/A	0
89	0.28	166	0.65	454	#N/A	0	#N/A	0	#N/A	0	#N/A	0
90	0.04	56	#N/A	0	#N/A	0	#N/A	0	#N/A	0	#N/A	0
91	1.57	56	#N/A	0	#N/A	0	#N/A	0	#N/A	0	#N/A	0
**92**	**1.25**	**832**	**5.32**	**766**	**5.32**	**801**	**5.32**	**987**	**#N/A**	**0**	**#N/A**	**0**
93	2.99	65	#N/A	0	#N/A	0	#N/A	0	#N/A	0	#N/A	0
94	1.77	50	1.11	64	0.95	192	#N/A	0	#N/A	0	#N/A	0
95	4.34	2962	5.09	5334	5.12	5859	#N/A	0	#N/A	0	#N/A	0
96	0.88	23	1.83	66	#N/A	0	#N/A	0	#N/A	0	#N/A	0
**97**	**0.01**	**55**	**1.47**	**59**	**1.46**	**63**	**4.79**	**1033**	**4.53**	**966**	**#N/A**	**0**
**98**	**0.00**	**205**	**3.06**	**427**	**3.12**	**571**	**#N/A**	**0**	**#N/A**	**0**	**#N/A**	**0**

Table B Descriptions of the selected suitable sites to generate the migration networks. The area is the sum of water and grass landscapes (km^2) in 1992 and 2012, respectively; the location is the country of the site; the type (B, S or W) represents breeding, stopover or wintering sites; the change ratio indicates the ratio of habitat loss; and the scenario indicates in which scenario the site was removed.

ID	Lon	Lat	1992			2012			Location	Type	Change ratio	Scenario
			Water	Grass	Area	Water	Grass	Area				
1	134.34	69.12	110.17	43.07	153.24	120.39	61.91	182.30	Russia	B	0.19	-
2	143.12	68.73	291.73	312.79	604.52	300.37	300.69	601.06	Russia	B	-0.006	50%
3	143.82	67.68	1359.46	1062.32	2421.78	1368.56	1441.55	2810.10	Russia	B	0.16	-
4	153.11	67.55	898.27	363.06	1261.33	836.71	373.20	1209.91	Russia	B	-0.041	40%
5	159.34	66.49	238.65	857.38	1096.02	238.65	913.08	1151.72	Russia	S	0.051	-
6	151.12	66.35	612.20	128.00	740.19	597.07	132.33	729.41	Russia	S	-0.015	50%
7	156.41	65.87	3.03	165.47	168.49	3.03	145.69	148.71	Russia	S	-0.117	20%
8	160.35	65.23	40.44	169.51	209.95	40.44	175.21	215.66	Russia	S	0.027	-
9	138.72	64.86	52.89	196.09	248.98	52.89	415.81	468.70	Russia	S	0.883	-
10	152.39	64.67	226.31	236.51	462.81	233.78	174.22	408.00	Russia	S	-0.118	10%
11	161.51	64.38	43.36	706.82	750.18	43.36	766.75	810.11	Russia	S	0.08	-
12	153.94	63.94	104.55	74.06	178.61	107.84	71.68	179.52	Russia	S	0.005	-
13	149.93	63.41	4.82	80.27	85.09	4.82	165.39	170.21	Russia	S	1	-
14	147.82	63.06	200.93	374.67	575.61	203.08	753.63	956.70	Russia	S	0.662	-
15	153.98	63.06	2.81	129.55	132.36	2.81	162.04	164.85	Russia	S	0.246	-
16	152.48	62.53	114.80	100.40	215.20	123.31	139.53	262.84	Russia	S	0.221	-
17	149.28	62.07	298.28	91.51	389.80	294.35	139.45	433.80	Russia	S	0.113	-
18	159.83	61.89	160.57	1762.10	1922.67	160.49	1840.15	2000.64	Russia	S	0.041	-
19	154.49	61.50	15.93	120.90	136.83	15.93	148.47	164.40	Russia	S	0.201	-

20	140.37	61.09	423.84	629.11	1052.96	424.73	641.10	1065.83	Russia	S	0.012	-
21	149.30	60.50	966.05	2950.12	3916.16	963.26	3862.47	4825.73	Russia	S	0.232	-
22	143.44	60.47	16.94	102.43	119.38	16.94	131.34	148.29	Russia	S	0.242	-
23	145.07	60.21	45.61	104.60	150.21	45.61	109.21	154.82	Russia	S	0.031	-
24	143.58	59.58	218.39	1275.57	1493.96	226.42	1345.99	1572.41	Russia	S	0.053	-
25	160.51	59.48	19.35	61.19	80.54	19.35	76.44	95.80	Russia	S	0.189	-
26	136.09	58.94	0.62	18.04	18.66	0.62	16.65	17.27	Russia	S	-0.075	30%
27	159.43	58.64	33.27	1069.90	1103.17	33.27	1112.91	1146.18	Russia	S	0.039	-
28	161.71	57.64	58.91	136.24	195.15	58.91	127.49	186.40	Russia	S	-0.045	30%
29	158.70	57.42	53.83	1533.52	1587.34	53.08	1491.71	1544.79	Russia	S	-0.027	40%
30	131.84	57.37	146.09	28.19	174.28	146.09	24.95	171.04	Russia	S	-0.019	40%
31	156.42	56.63	248.15	4604.62	4852.77	244.91	4655.88	4900.79	Russia	S	0.01	-
32	135.56	56.53	45.13	294.80	339.93	45.13	360.68	405.80	Russia	S	0.194	-
33	160.87	56.43	399.81	629.30	1029.11	397.72	584.52	982.24	Russia	S	-0.046	30%
34	130.58	55.95	109.93	61.46	171.38	109.93	89.72	199.65	Russia	S	0.165	-
35	159.55	55.71	43.23	112.74	155.97	43.23	96.17	139.40	Russia	S	-0.106	20%
36	156.89	55.41	5.27	365.68	370.95	5.27	359.55	364.82	Russia	S	-0.017	50%
37	159.74	54.88	280.16	267.96	548.12	280.16	280.78	560.94	Russia	S	0.023	-
38	155.78	54.54	124.30	2486.05	2610.35	124.30	2590.38	2714.68	Russia	S	0.04	-
39	127.93	54.34	2340.87	646.24	2987.11	2408.88	695.90	3104.77	Russia	S	0.039	-
40	134.18	54.33	54.76	136.05	190.82	54.76	59.85	114.61	Russia	S	-0.399	5%
41	132.70	54.04	14.67	92.50	107.17	14.67	77.65	92.32	Russia	S	-0.139	10%
42	134.91	53.83	0.46	53.13	53.59	0.46	42.80	43.25	Russia	S	-0.193	10%
43	139.91	53.46	565.38	979.05	1544.44	619.75	1216.12	1835.87	Russia	S	0.189	-
44	158.06	53.11	93.68	221.28	314.96	93.44	236.51	329.95	Russia	S	0.048	-
45	136.39	52.76	14.80	915.70	930.50	14.75	819.85	834.60	Russia	S	-0.103	20%
46	156.58	52.29	428.23	1535.52	1963.76	422.88	1588.87	2011.75	Russia	S	0.024	-

47	140.00	51.81	1220.97	4397.77	5618.74	1457.58	5155.52	6613.10	Russia	S	0.177	-
48	136.55	51.77	589.15	2953.73	3542.88	597.21	2861.09	3458.30	Russia	S	-0.024	40%
49	127.88	50.54	447.56	14007.32	14454.88	458.43	12995.45	13453.87	Russia	S	-0.069	30%
50	119.25	50.36	18.47	3455.68	3474.15	18.47	3523.85	3542.32	China	S	0.02	-
51	115.78	50.02	1073.00	7833.93	8906.94	552.11	8040.41	8592.52	Russia	S	-0.035	40%
52	129.66	49.45	228.82	7303.70	7532.52	238.54	7009.19	7247.73	Russia	S	-0.038	40%
53	123.73	49.25	2.57	1799.92	1802.49	2.57	1673.32	1675.89	China	S	-0.07	30%
54	135.82	49.19	2891.74	25034.92	27926.66	3206.14	24557.54	27763.68	Russia	S	-0.006	50%
55	114.43	48.64	86.35	430.16	516.51	63.17	411.26	474.43	Mongolia	S	-0.081	30%
56	115.47	48.45	1.71	330.08	331.79	1.71	325.08	326.79	Mongolia	S	-0.015	50%
57	126.35	48.18	212.31	21725.03	21937.34	247.91	21275.66	21523.56	China	S	-0.019	40%
58	132.70	47.92	395.87	7745.98	8141.85	472.08	7672.08	8144.15	China	S	0	-
59	118.04	47.63	3968.63	44776.57	48745.20	3413.28	46352.38	49765.66	Mongolia	S	0.021	-
60	142.73	47.44	45.58	1.61	47.19	45.58	0.99	46.57	Russia	S	-0.013	50%
61	143.00	46.69	403.45	27.52	430.96	402.64	28.43	431.07	Russia	S	0	-
62	131.68	45.96	5412.48	39431.63	44844.10	5481.05	39660.91	45141.96	China	S	0.007	-
63	134.71	45.90	8.97	392.93	401.90	8.97	579.52	588.48	Russia	S	0.464	-
64	141.94	45.19	65.04	19.49	84.53	64.35	22.00	86.35	Japan	S	0.022	-
65	123.72	44.55	5253.08	173233.28	178486.36	5839.24	171488.44	177327.68	China	S	-0.006	50%
66	128.64	43.74	152.78	3644.38	3797.16	160.84	4031.58	4192.42	China	S	0.104	-
67	122.82	43.63	21.23	2134.55	2155.78	21.23	2094.24	2115.47	China	S	-0.019	40%
68	116.70	43.28	244.54	3868.92	4113.46	245.26	3851.95	4097.21	China	S	-0.004	50%
69	131.98	43.27	138.44	349.70	488.14	136.05	398.07	534.12	Russia	S	0.094	-
70	142.67	43.25	993.08	506.02	1499.10	996.83	549.19	1546.02	Japan	S	0.031	-
71	143.54	42.60	54.18	33.54	87.71	54.18	34.23	88.41	Japan	S	0.008	-
72	130.64	42.54	228.42	1071.85	1300.28	231.58	1241.68	1473.27	Russia	S	0.133	-
73	120.74	41.06	24.95	2861.39	2886.33	23.88	2756.12	2779.99	China	S	-0.037	40%

74	122.69	40.99	690.41	4993.93	5684.34	622.64	4988.95	5611.59	China	S	-0.013	50%
75	119.74	40.73	6.26	1198.03	1204.29	6.26	1201.16	1207.42	China	S	0.003	-
76	115.72	40.34	61.24	3170.86	3232.10	45.61	2906.30	2951.91	China	S	-0.087	20%
77	124.98	40.09	2530.32	39241.56	41771.88	2519.45	42828.90	45348.35	North Korea	S	0.086	-
78	119.23	39.78	134.21	2296.14	2430.35	111.99	2106.42	2218.41	China	S	-0.087	20%
79	127.47	39.77	92.48	1254.61	1347.09	91.35	1345.14	1436.49	North Korea	S	0.066	-
80	117.65	39.14	2700.42	21360.85	24061.27	2537.73	19340.99	21878.73	China	S	-0.091	20%
81	139.92	38.85	4.87	36.03	40.90	4.87	34.21	39.08	Japan	S	-0.045	30%
82	140.27	38.21	1322.17	2350.77	3672.94	1352.76	1335.71	2688.48	Japan	S	-0.268	5%
83	118.90	37.92	481.87	1465.21	1947.08	530.80	1397.36	1928.16	China	S	-0.01	50%
84	115.57	37.58	35.87	1894.41	1930.27	35.92	1725.27	1761.19	China	S	-0.088	20%
85	116.08	36.10	185.49	2428.82	2614.31	211.48	2039.00	2250.48	China	W	-0.139	10%
86	127.72	35.95	1285.23	23804.18	25089.41	1255.42	26494.69	27750.11	South Korea	W	0.106	-
87	136.83	35.65	1098.06	1699.68	2797.74	1090.86	799.80	1890.66	Japan	W	-0.324	5%
88	133.07	35.44	205.27	44.81	250.08	204.87	19.22	224.09	Japan	W	-0.104	20%
89	110.61	34.85	61.19	2853.87	2915.06	90.12	2512.95	2603.07	China	W	-0.107	20%
90	135.28	34.70	34.85	286.24	321.09	32.01	135.41	167.42	Japan	W	-0.479	5%
91	134.10	34.35	342.66	784.36	1127.02	322.48	387.60	710.08	Japan	W	-0.37	5%
92	120.24	34.23	252.06	182.17	434.23	265.28	149.38	414.66	China	W	-0.045	40%
93	130.70	33.46	315.04	424.24	739.28	311.29	311.35	622.64	Japan	W	-0.158	10%
94	114.24	33.03	60.22	62.63	122.86	78.08	36.96	115.04	China	W	-0.064	30%
95	118.23	32.96	21414.63	42342.29	63756.92	20521.65	37730.72	58252.37	China	W	-0.086	30%
96	131.27	32.01	73.61	60.44	134.05	73.53	46.47	119.99	Japan	W	-0.105	20%
97	112.38	30.43	395.26	28.48	423.74	395.74	26.82	422.56	China	W	-0.003	50%
98	112.77	29.27	3827.68	1004.05	4831.73	3169.12	1418.34	4587.46	China	W	-0.051	30%

Table C Basic network metrics of the six theoretical fall migration networks.

Network scenarios	Site number	Link number	Link density	Distance N-S (km)*
Complete network	98	2356	0.50	4645
Removed 10% sites	88	1922	0.50	4645
Removed 20% sites	78	1571	0.52	4645
Removed 30% sites	68	1264	0.55	4527
Removed 40% sites	58	931	0.56	4527
Removed 50% sites	47#	670	0.62	3714

* Distance N-S represents the geographic distance between the northernmost and southernmost sites.

\# After the removal of 40% sites with habitat loss, we aim to remove all the rest sites with habitat loss in next scenario, thus we removed 11 sites (one more site compared to the removal in previous scenario) to generate the scenarios of removed 50% sites.

Table D Functional connectivity of each network scenario, and the corresponded estimation of population sizes.

Network scenarios	Functional connectivity[$]	Estimated population size[*]
Complete network	306730	10000
Removed 10% sites	305146	9956
Removed 20% sites	295189	9680
Removed 30% sites	275102	9124
Removed 40% sites	257923	8649
Removed 50% sites	114592	4680

[$] The functional connectivity was measured by the index of "equivalent connected area" using a directed graph theory algorithm [9]. The detailed method was described by Xu et al., 2019 [8].

[*] The population sizes were estimated by implementing a modified regression relationship between ratio of population change (RPC) and ratio of functional connectivity change (RFCC) (RPC = 0.216 * RFCC) [8].

Reference

1. Kölzsch A, Müskens GJDM, Kruckenberg H, Glazov P, Weinzierl R, Nolet BA, et al. Towards a new understanding of migration timing: slower spring than autumn migration in geese reflects different decision rules for stopover use and departure. Oikos. 2016;125: 1496–1507. doi:10.1111/oik.03121
2. Deng X, Zhao Q, Fang L, Xu Z, Wang X, He H, et al. Spring migration duration exceeds that of autumn migration in Far East Asian Greater White-fronted Geese (Anser albifrons). Avian Res. 2019;10: 1–11. doi:https://doi.org/10.1186/s40657-019-0157-6
3. R Development Core Team. R: A language and environment for statistical computing. R Found Stat Comput Vienna Austria. 2016. doi:10.1038/sj.hdy.6800737
4. Impe J van. Long-term reproductive performance in White-fronted Geese Anser a. albifrons and Tundra Bean Geese A. fabalis rossicus wintering in Zeeland (The Netherlands). Bird Study. 1996;43: 280–289. https://doi.org/10.1080/00063659609461020
5. Schock WG, Fischer JB, Ely CR, Stehn RA, Welker JM, Causey D. Variation in age ratio of midcontinent greater white-fronted geese during fall migration. J Fish Wildl Manag. 2018;9: 340–347. doi:10.3996/112015-JFWM-117
6. Cunningham SA, Zhao Q, Weegman MD. Increased rice flooding during winter explains the recent increase in the Pacific Flyway White-fronted Goose Anser albifrons frontalis population in North America. Ibis (Lond 1859). 2021;163: 231–246. doi:10.1111/ibi.12851
7. Hénaux V, Samuel MD, Bunck CM. Model-based evaluation of highly and low pathogenic avian influenza dynamics in wild birds. PLoS One. 2010;5: e10997. doi:10.1371/journal.pone.0010997
8. Xu Y, Si Y, Wang Y, Zhang Y, Prins HHT, Cao L, et al. Loss of functional connectivity in migration networks induces population decline in migratory birds. Ecol Appl. 2019;29: e01960. doi:10.1002/eap.1960
9. Saura S, Bodin Ö, Fortin M. Stepping stones are crucial for species' long-distance dispersal and range expansion through habitat networks. J Appl Ecol. 2014;51: 171–182. doi: 10.1111/1365-2664.12179
