# Supplementary material for: Habitat loss exacerbates pathogen spread: An Agent-based model of avian influenza infection in migratory waterfowl
Source: PLoS Comput Biol. 2022 Aug 18;18(8):e1009577. doi: 10.1371/journal.pcbi.1009577 (PMC9426877; doi:10.1371/journal.pcbi.1009577)
Supplement: S2 Appendix — (ZIP) [file pcbi.1009577.s002.zip › README_Netlogo.docx]

This model belong to the manuscript:

**Habitat loss exacerbates pathogen spread: An Agent-based model of avian influenza infection in migratory waterfowl**

Shenglai Yin^1,3^, Yanjie Xu^2,3^, Mingshuai Xu^1^, Mart C.M. de Jong^4^, Mees R.S. Huisman^3^, Andrea Contina^5^, Herbert H. T. Prins^6^, Zheng Y. X. Huang^1*^, Willem F. de Boer^3^

^1^ College of Life Science, Nanjing Normal University, Nanjing, China

^2^ The Finnish Museum of Natural History, University of Helsinki, Helsinki, Finland

^3^ Wildlife Ecology and Conservation Group, Wageningen University, Wageningen, The Netherlands

^4^ Quantitative Veterinary Epidemiology Group, Wageningen University, Wageningen, The Netherlands

^5^ Department of Microbiology and Plant Biology, Center for Earth Observation and Modeling, University of Oklahoma, Norman, Oklahoma, USA

^6^ Department of Animal Sciences, Wageningen University, Wageningen, The Netherlands

*Corresponding author:

Dr. Zheng Y.X. Huang

Email: zhengyxhuang@gmail.com (ZXYH)

For technical support:

Dr. Shenglai Yin

Email: shenglai.yin@outlook.com (SLY)

First and foremost, this Netlogo model had to be placed in the exact location with the supporting files. The supporting files are 1) the “*word-shape-files*” folder, 2) *node_list.txt* files, and 3) *edge_list.txt* files.

This model simulates the fall migration of the Greater white-fronted goose in the East Asian-Australasian Flyway (EAAF). We aim to test the effects of habitat loss on goose migration and, more importantly, the spread and transmission of the avian influenzas virus (AIV).

The migration network was pre-generated via network analysis (see main text) and imported into the Netlogo scenario by scenario.

The driving mechanism of goose movement was inspired by: Caz Taylor et al., 2016 The response of migratory populations to phenological change: A Migratory Flow Network modeling approach.

The mechanism of SIR transmission was modified from Yin et al., 2020 Effects of migration network configuration and migration synchrony on infection prevalence in geese.

We keep the model as simple as possible and aim to simulate fall migration only. But it is possible to simulate spring migration if the spring network (northward linked) was pre-generated and imported into the Netlogo model. Moreover, the model can test other theories and/or variables by adding new components.

For technical help and collaboration, please contact:

Dr. Shenglai Yin (shenglai.yin@outlook.com)

Dr. Zheng XY Huang (zhengyxhuang@gmail.com)
